# The development of a data dictionary with clinical variables for artificial intelligence-driven tools in research on abdominal aortic aneurysms and peripheral arterial disease

**DOI:** 10.1093/ehjdh/ztaf091

**Published:** 2025-08-20

**Authors:** Lotte Rijken, Sabrina L M Zwetsloot, Catelijne Muller, Marlies P Schijven, Vincent Jongkind, Kak Khee Yeung, VASCUL-AID consortium, Igor Koncar, Ivan Tomic, Marina Dias-Neto, Katarzyna D Bera, Riikka Tulamo, Maarit Venermo, Mirjami Laivuori, Christian-Alexander Behrendt, Stefan P M Smorenburg, Corrette Ploem, Roosmarie Jessen, Bert-Jan H van den Born, Ronak Delewi, Jelmer Wolterink, Noeska Smit, Ivana Išgum, Henk A Marquering, Fabio Catarinella, Fabien Lareyre, Juliette Raffort, Maja Živković, Tamara Djuric, Aleksandra Stankovic, VASCUL-AID collaborators, Rupert Bauersachs, Manar Khashram

**Affiliations:** Amsterdam UMC, Location University of Amsterdam, Department of Surgery, Meibergdreef 9, 1105AZ Amsterdam, the Netherlands; Amsterdam Cardiovascular Sciences, Atherosclerosis and Ischemic Syndromes, Amsterdam, The Netherlands; Amsterdam Public Health, Digital Health, Amsterdam, the Netherlands; Amsterdam UMC, Location University of Amsterdam, Department of Surgery, Meibergdreef 9, 1105AZ Amsterdam, the Netherlands; Amsterdam Cardiovascular Sciences, Atherosclerosis and Ischemic Syndromes, Amsterdam, The Netherlands; ALLAI, Amsterdam, the Netherlands; Amsterdam UMC, Location University of Amsterdam, Department of Surgery, Meibergdreef 9, 1105AZ Amsterdam, the Netherlands; Amsterdam Public Health, Digital Health, Amsterdam, the Netherlands; Amsterdam Gastroenterology and Metabolism, Amsterdam, the Netherlands; Amsterdam UMC, Location Vrije Universiteit Amsterdam, Department of Surgery, De Boelelaan, 1105, 1081HV Amsterdam, the Netherlands; Amsterdam Cardiovascular Sciences, Microcirculation, Amsterdam, the Netherlands; Amsterdam UMC, Location University of Amsterdam, Department of Surgery, Meibergdreef 9, 1105AZ Amsterdam, the Netherlands; Amsterdam Cardiovascular Sciences, Atherosclerosis and Ischemic Syndromes, Amsterdam, The Netherlands

**Keywords:** Abdominal aortic aneurysm, Peripheral arterial disease, Clinical data dictionary, Artificial intelligence, Expert consensus

## Abstract

**Aims:**

Patients with abdominal aortic aneurysms and peripheral arterial disease (arterial vascular diseases) carry a high disease burden and are likely to experience cardiovascular events. Novel strategies using artificial intelligence could identify which patients with arterial vascular diseases are at high risk of cardiovascular disease progression. Structured data dictionaries are needed to ensure high-quality, unbiased, and ethically sound data input for artificial intelligence models. The aim of this study was to obtain expert consensus-based data dictionaries that adhere to applicable ethical guidelines to support research on arterial vascular diseases.

**Methods and results:**

The data dictionaries were created through a modified Delphi approach to achieve consensus among key opinion leaders in the cardiovascular field. First, data requirements were defined and variable longlists were created per disease through a literature review. Secondly, written feedback rounds were held. Lastly, face-to-face meetings were held to establish consensus on the final data dictionaries. During the whole process, ethical and legal experts on trustworthy artificial intelligence were involved to ensure adherence to corresponding guidelines and laws. The aneurysm data dictionary contains 312 variables, and the peripheral arterial disease data dictionary contains 325 variables. A total of 16 clinical experts were involved in the creation, including 12 vascular surgeons, two vascular medicine specialists, one cardiologist, and one gastroenterology surgeon and digital health expert.

**Conclusion:**

Two expert consensus-based data dictionaries for use in clinical and artificial intelligence research on arterial vascular diseases were created, developed for application in research on predicting disease progression and cardiovascular risk.

## Graphical Abstract

**Graphical Abstract:**
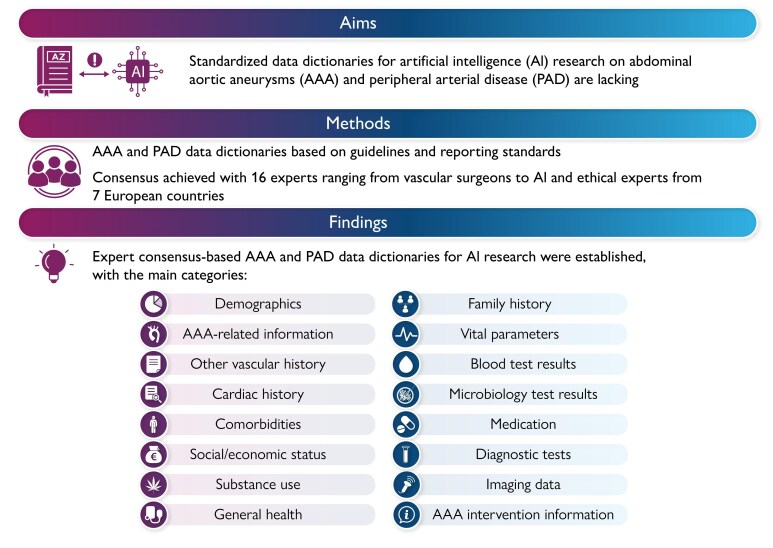

## Introduction

Peripheral arterial disease (PAD) and abdominal aortic aneurysms (AAA) are diseases of the arterial vasculature, primarily caused by atherosclerosis, which contribute to a high global burden of disease.^1^ Prevalence of PAD is increasing due to an ageing population.^2^ Moreover, patients with PAD and AAA are at higher risk of developing major adverse cardiovascular events than the general population.^3–5^ Some risk factors, in particular lifestyle-related factors (smoking, decreased exercise, unhealthy diet), genetics, and comorbidities (hypertension, hypercholesterolaemia), are known to be associated with PAD and AAA and may cause cardiovascular disease or promote ongoing cardiovascular disease progression.^6–8^ Despite current recommendations for cardiovascular risk management, disease progression is still prominent.^8,9^ This emphasizes the need for development of novel strategies to identify which AAA and PAD patients are at high risk of cardiovascular disease progression and for whom intervention could be effective.

One promising development is the use of artificial intelligence (AI) to aid in clinical decision making and gaining insight into disease aetiology and progression. Artificial intelligence enables computer systems to simulate human intelligence and is able to learn from historical data and make predictions.^10^ The past decade has seen a rise in the use of AI in clinical research in various applications due to its ability to process large quantities of data effectively.^11,12^ This allows for relatively rapid identification of correlations in extensive datasets (popularly known as ‘big data’) that are too large or complex to be dealt with by traditional data-processing software. When developing an AI model, only high-quality data should be provided to build a reliable model.^13^ This is especially challenging in cases where medical data are retrieved from electronic health record resources, which are bound to be heterogenous in format.^11^ For this reason, structured data collection, using a structured data repository and data management plan, is needed.

When training data are of poor quality, biased, and ethically unsound, or otherwise contain incorrect or misleading information, the AI model will learn and potentially reinforce those ingrained biases and/or inaccuracies.^14,15^ If such an AI model would be applied in the healthcare domain, this could adversely affect patients. This may potentially jeopardize their fundamental rights as enshrined in the EU Charter of Fundamental Rights (ECFR), such as the rights to life (Art. 2 ECFR) and non-discrimination (Art. 21 ECFR). Aimed at protecting these and other fundamental rights, the EU Ethics Guidelines for Trustworthy AI identified key requirements for Trustworthy AI.^16^ The EU AI Act (2024) was built on these key requirements, laying down stringent regulatory requirements and obligations for training, testing, and validating data used in ‘high-risk’ AI systems.^17^ The importance of carefully selecting which variables to include in the AI model cannot, therefore, be understated and must be respected.

In literature, reporting standards have been created in an attempt to homogenize definitions and outcomes in the domain of vascular surgery.^18–23^ While these reporting standards may be used as guidance for the development of AI tools, they did not consider the EU Ethics Guidelines for Trustworthy AI as these were generated prior to publication of these standards. Moreover, these standards were developed with registries or clinical trials in mind; hence, they may not be applicable in studies using ‘big data’ or AI applications, since these require ‘data dictionaries’. Data dictionaries for use in clinical AI applications are comprehensive lists of variables known to be related to the disease, which also consider social as well as clinical factors potentially related to the disease, while reporting standards include only factors known to be related to the disease.

To our knowledge, no input variable standards have yet been developed for use in the development of AI models to enable reliable clinical predictions for patients with PAD and AAA who do not (yet) require an intervention. The aim of this study was to design data dictionaries fit for use in the VASCUL-AID studies to collect electronic health record (EHR) data to standardize (AI) research on AAA and PAD to ensure completeness and to minimize selection bias. Artificial intelligence has the potential to find meaningful correlations in an extensive dataset, identifying novel risk factors (from demographic information to laboratory values) related to disease progression. This is especially important since disease progression in individual patients cannot be accurately predicted with known risk factors. Risk factors of exceptional interest are those that are modifiable, such as lifestyle factors. The data dictionaries have been developed for use in retrospective EHR data collection and as a basis for EHR data collection in prospective studies, for which patient-reported outcomes (PROs) and patient-reported outcome measures (PROMs) are recommended to be added. The VASCUL-AID project is a European collaboration project of 14 centres, which aims to establish an AI-driven platform including a mobile health application to predict cardiovascular disease progression.^24^ Ethical and legal experts were involved to ensure adherence to the key requirements of the EU AI Act and other relevant regulations. The data dictionaries can also be used in other cardiovascular AI prediction models based on EHR data for preoperative patients with PAD and AAA.

## Methods

The expert consensus-based data dictionaries were created in a stepwise manner. A modified Delphi approach was employed to achieve consensus among key opinion leaders (KOLs) in the cardiovascular field.^25^ The Delphi methodology is an established method of attaining consensus among participants through indirect and anonymous feedback and has been used before in the creation of vascular surgery reporting standards.^21–23^ First, data requirements were defined, and longlists of variables were created per disease on the basis of a literature review on reporting standards and (inter)national guidelines. Following this, written feedback rounds by an expert consensus group took place. Lastly, face-to-face meetings were held with KOL to facilitate discussion on in- and exclusion of selected variables, to form the final data dictionaries.

### Expert consensus group

The authors L.R. and S.L.M.Z., both vascular surgery PhD researchers, created the initial variable lists based on a literature search and moderated the consensus meetings. The expert consensus group consisted of KOLs in the cardiovascular field; all 18 VASCUL-AID clinical consortium partners and two additional external specialists in the field of vascular surgery were invited to participate. Consensus was established through face-to-face discussions, in which a group size of 6–12 participants was aimed for to promote balanced discussion and prevent dominance by individuals.^25^ Furthermore, the development of the data dictionaries was overseen by experts in the field of AI and ethics, who ensured that all applicable regulations were complied with. Participant recruitment was limited to experts, and patients were excluded from participation, since sufficient knowledge of factors involved in disease progression was required to establish consensus.

### End users

The intended end users of the data dictionaries are researchers who employ big data and/or AI technologies in studies on patients with AAA or PAD using EHR data collection methods.

### Definition of patients

The patient population of the to-be-developed VASCUL-AID mobile health application (app) includes patients of both sexes, aged 40–90 with either lower extremity PAD (atherosclerotic obstruction from the aortoiliac segments to the pedal arteries) and/or with an AAA of at least 30 mm, currently under surveillance by a vascular surgeon. A diagnosis of PAD or AAA by a vascular surgeon is required for inclusion. A diagnosis of PAD is defined as ABI lower than or equal to 0.9 and/or radiologic confirmation of stenosis that causes a patient’s clinical complaints, as assessed by a vascular surgeon.^26^

### Data dictionary structure

Data collection timepoints were defined to structure the variable list and to facilitate the process of future data collection. As patient information may change over time, the variable list was divided into two types of data collection timepoints: information reported at the time of an ‘index’ (or: first) visit with a vascular surgeon and information reported at the time of a ‘follow-up visit’. Variables were categorized according to different medical topics: demographics, clinical information related to AAA and/or PAD, other vascular history, comorbidities, socioeconomic status, smoking status and substance use, general health, family history, vital parameters, blood test results, microbiology test results, medication, diagnostic test, imaging data, AAA and PAD intervention parameters, and AAA rupture parameters. In future applications, these variables should be collected at the abovementioned data collection timepoints. A detailed explanation of the data collection timepoints and definitions can be found on the first page of the data dictionaries attached in Supplementary material online, *Tables S1* and *S2*.

### Ethics and legal considerations

In developing the data dictionaries, the Health Ethical, Legal and Societal Implications (HELSI) framework (unpublished) developed within the VASCUL-AID project by ethical and legal experts was used and respected. This HELSI-AI framework, based on the EU Ethics Guidelines for Trustworthy AI,^16^ identifies the main legal and ethical norms and key principles in the application of AI prediction tools in healthcare and guides trustworthy data collection and development of AI models in the VASCUL-AID project. The principles ‘Human Agency and Oversight’, ‘Data and Data Governance’, ‘Transparency’, ‘Diversity, Non-discrimination and Fairness’, and ‘Accountability’ are relevant to be taken into account when selecting data variables.

### Variable selection

The following main principles for the data dictionaries were formulated by the study management group:

1. Relevance: including variables that are used in clinical practice.
2. Conciseness to maintain simplicity.
3. Comprehensibility: definitions are added.
4. Transparency: a rationale is included to explain the reasoning.
5. Completeness: all relevant variations are covered within variables.
6. Non-ambiguous: there is no overlap in meaning between variables.

Moreover, as AI research aimed at predicting future patient outcomes relies on and allows for the analysis of large volumes of data, variables for which the medical community is unsure if they are directly associated with PAD or AAA must be included as well because they *might* be associated with PAD or AAA, or one of their known predictors.

**Figure 1:**
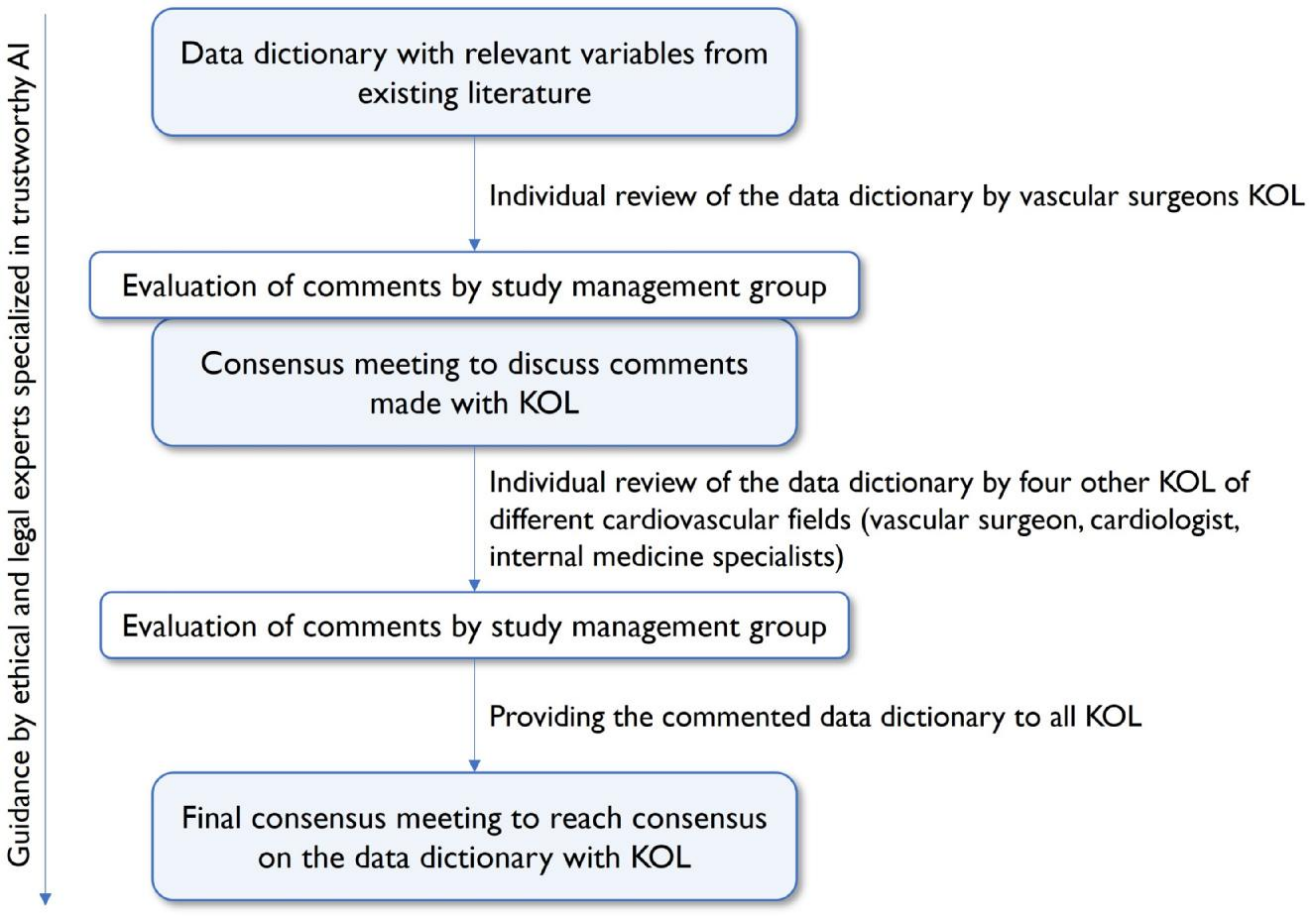
Flowchart variable selection procedure. KOL, key opinion leaders; AI, artificial intelligence.

In *Figure 1* a flowchart with all steps performed to obtain the data dictionaries is visualized. First, a variable longlist was created based on a literature review. Literature was searched for reporting standards (including those made for use in vascular registries) and the most recent society reporting guidelines on PAD and AAA until November 2023 (when the data dictionary was finalized).^18–23^ Well-known and validated clinical classification systems, such as the Clavien–Dindo classification, were used for objective reporting of variables where applicable.^27^ Disease- and general health-related variables were retrieved and deduplicated by L.R. Variables and answer options may differ between countries, among them socioeconomic indicators (e.g. educational level); therefore, these variables are generalized for application across healthcare systems. If harmonization is not possible, an ‘other’ category is provided. Socioeconomic indicators, where available, will be extracted from EHRs. Patient-reported outcomes such as quality of life were not measured since it was not feasible to retrieve these from EHR data repositories. For prospective data collection studies, obtaining health-related quality of life information is feasible and should be collected.

Imaging variables were limited, because imaging analysis and data extraction are performed separately in the VASCUL-AID project and are still under development.^28^ Nonetheless, imaging variables reported in the medical file such as AAA diameter are already included in the current dictionary. Additional imaging parameters, such as other anatomical and haemodynamic parameters, can be obtained after application of (automated) image analysis tools. These image analyses will result in new variables (tabular data format) that can be added to the other variables in the data dictionary. In the retrospective VASCUL-AID study, we will first analyse the clinical dataset and imaging separately, to determine the predictive power of each of these modalities. In the second part of this study, the clinical and imaging data will be combined in the same predictive model to obtain a multimodal risk prediction.

Definitions with corresponding references were retrieved from society guidelines, if possible, and otherwise from reporting standards or other existing literature. Rationales for inclusion in the data dictionaries were defined for all variables, if possible, on the basis of reporting standards or existing literature or otherwise from discussion among the study management group.^2,9,18,22,26,29–48^ All definitions and rationales were retrieved, defined, and cross-checked by L.R. and S.L.M.Z., and discrepancies were discussed between them to reach consensus. Disagreements and the addition of potentially other relevant variables were discussed in study management group meetings. Potentially relevant variables were mostly comorbidities (primarily cardiac, but also many other comorbidities), variables related to interventions, and socioeconomic indicators. While several of these variables may have been investigated in the past, they have not been studied in a dataset as extensive as those of the retrospective studies, and they have not been analysed through machine learning. Ethical and legal experts were involved throughout the entire study to ensure compliance with the HELSI-AI framework, EU Ethics Guidelines for Trustworthy AI, and the EU AI Act.

### First review of the data dictionary

The variable longlist was distributed to all KOLs for them to individually appraise the variable list on whether the definitions were correct and rationales were logical and if there were any missing variables. Variables were excluded if they were considered redundant and were impossible to collect from retrospective EHRs. The study management group reviewed all comments and made minor changes (addressing spelling errors and wording suggestions) to the variable longlist accordingly. Suggestions for definition or rationale changes, the addition or removal of outcomes, and other discussion points were considered major and were therefore discussed in a consensus meeting.

### First consensus meeting

Following written review of the variable lists, a face-to-face meeting was organized to discuss and establish consensus on all remaining discussion points of the PAD and AAA variable lists. The first consensus meeting took place during the European Society for Vascular Surgery (ESVS) Annual Meeting on 26 September 2023. Consensus was pre-defined as follows:

1. Greater than 75% agreement: consensus on including or excluding the parameter or changing the rationale/definition according to the suggested comment.^49^ No further discussion is required.
2. Fifty to seventy-five per cent agreement will provoke a short discussion, after which another vote will ensue. If, after the second vote, there is still 50–75% consensus, the study management group will consult the literature, and the discussion point will be discussed in the next consensus meeting.

During the consensus meeting, the discussion points, based on the first review round, and corresponding variables were shown. An opportunity was provided for further comments and inquiries, after which agreement was assessed by voting.

### Second review of the data dictionary

After the first consensus meeting, the changes on which consensus was established were processed in the variable lists. The changes were highlighted, and comments were placed in case of definition changes and discussion points. These updated variable lists were provided to four KOLs via e-mail: two VASCUL-AID consortium partners (a cardiologist and vascular medicine specialist), one external reviewer, and one member of the project’s Advisory Board who had not seen the variable longlist before. The vascular internal medicine specialist and cardiologist added variables to the list relating to their respective discipline. The purpose of this was to obtain feedback on the variable lists from KOLs from relevant fields other than vascular surgery and from vascular surgeons from outside of the VASCUL-AID consortium. These reviewers were given 2 weeks to review the variable lists. Changes and/or comments were marked in the variable lists and sent to all VASCUL-AID KOLs to review before the final consensus meeting.

### Final consensus meeting

The final consensus meeting was held online and was organized to discuss the feedback from the second review round and resolve final inconsistencies. This meeting was held through Microsoft Teams on 2 November 2023.^50^ Consensus was pre-defined as follows:

1. Greater than 75% agreement: consensus on including or excluding the parameter or changing the rationale/definition according to the suggested comment. No further discussion is required.
2. Fifty to seventy-five per cent agreement will provoke a short discussion, after which another vote will ensue. If, after the second vote >50% consensus is obtained, the discussion continues until >75% agreement is reached.

Following the final expert consensus meeting, the data dictionaries were finalized according to the decisions made in the final consensus meeting. The finalized data dictionaries were distributed to all consortium members.

## Results

### Variable selection

The variable lists were structured into index visit with vascular surgeon, follow-up visit, events, and repeated measurements and were subdivided into clinical categories, shown in *Table 1*. Categories are either disease-specific or encompass information about comorbidities and general health, but medical tests, social information, and family history are also included. Rationales, definitions, and supporting informative texts were added to the variables where necessary.

**Table 1:** Abdominal aortic aneurysm categories (*n* = 18) and peripheral arterial disease categories (*n* = 17) of the initial variable list

AAA categories	PAD categories
Demographics including ethnicity information	Demographics including ethnicity information
AAA-related information	PAD-related information
Other vascular history	Other vascular history
Cardiac history	Cardiac history
Comorbidities	Comorbidities
Social/economic status	Social/economic status
Substance use	Substance use
General health	General health
Family history	Family history
Vital parameters	Vital parameters
Blood test results	Blood test results
Microbiology test results	Microbiology test results
Medication	Medication
Diagnostic tests	Diagnostic tests
Imaging data	Imaging data
AAA intervention–related information	PAD intervention–related information
Mortality information	Mortality information
AAA rupture–related information	

AAA, abdominal aortic aneurysm; PAD, peripheral arterial disease.

### Consensus meetings

Eight KOLs commented on the variable longlist before the first consensus meeting. The first consensus meeting included six KOLs, and the final consensus meeting included eight KOLs. Baseline characteristics of participants are shown in *Table 2*. A total of 11 (69%) KOLs were vascular surgeons, and eight (50%) KOLs were female. During the consensus process, three meetings with the ethical and legal experts were held to ensure compliance with relevant AI regulations. Supplementary material online, *Table S3*, exhibits the variables that were discussed during the consensus meetings and the rationale behind including, removing, or changing variables.

**Table 2:** Baseline characteristics of key opinion leaders participating in the consensus process

Occupation	Sex	Country of residency	Years of experience as specialist in respective field	Participated in meeting 1, meeting 2, or mailing
Vascular surgeon	F	NL	7	1, 2, mailing
Vascular surgeon	M	NL	8	2, mailing
Vascular surgeon	F	PT	6	1, 2, mailing
Vascular surgeon	M	DE	6	1, 2, mailing
Vascular surgeon	M	RS	6	1, 2, mailing
Vascular surgeon	M	RS	7	Mailing
Vascular surgeon	M	UK	6	Mailing
Vascular surgery registrar	F	UK	6	Mailing
Vascular surgeon	F	FI	24	2, mailing
Vascular surgeon	F	FI	7	2, mailing
Vascular surgeon	F	FI	4	1, Mailing
Vascular surgeon	M	NZ	9	Mailing
Vascular internal medicine specialist	M	DE	26	Mailing
Vascular internal medicine specialist	M	NL	20	Mailing
Cardiologist	F	NL	6	Mailing
Gastroenterology surgeon and expert in the field of digital health	F	NL	16	Mailing
EU policy and responsible AI expert	F	NL	25	Ensuring compliance with AI regulations
Professor in health law and health technology	F	NL	25	Ensuring compliance with ethics regulations

### Final data dictionary

Following the final consensus meeting, the data dictionaries for research on PAD and AAA were standardized as shown in Supplementary material online, *Tables S1* and *S2*, respectively. A total of 312 unique variables were included in the AAA dictionary, and 325 in the PAD dictionary.

## Discussion

This study has established two expert consensus-based data dictionaries within vascular surgery for use in clinical as well as AI research on PAD and AAA. They are developed for application in research on predicting disease progression and risk of cardiovascular events in patients with PAD and AAA. The AAA data dictionary contains 312 variables across 18 categories, and the PAD data dictionary contains 325 variables across 17 categories. A total of 18 experts were involved in the creation of the data dictionaries, of which 12 were vascular surgeons including endovascular specialists, two were vascular medicine specialists, one was a cardiologist, one was a gastroenterology surgeon and public health expert, and two were AI/ethics specialists.

To date, there is no definitive cure to prevent disease progression in patients with AAA and PAD. Existing reporting standards are not developed for ‘big data’ AI–driven clinical research, which require large quantities of data not only to find meaningful associations but also for translation to real-life clinical practice, in which an abundance of clinical and social factors is of influence.^14^ For example, in our data dictionaries, cardiac history is recorded in detail, and socioeconomic factors such as education level are considered as well. Additional factors we considered in regard to a clinically relevant and high-quality dataset are the applicability of a data dictionary in practice to prevent inaccuracies as much as possible. A well-thought standardized data dictionary is thus warranted, which the current study aimed to develop.

Strengths of this study include its European expert consensus based on existing society guidelines and reporting standards. Throughout development of the data dictionaries, clinical relevance, comprehensibility, transparency, and simplicity were pursued. The HELSI-AI framework was considered throughout the study—in particular, the relevant key requirements for trustworthy AI of the Ethics Guidelines for Trustworthy AI and the legal requirements and obligations regarding data relevance and data accountability of the AI Act. Face-to-face meetings with KOLs from seven European countries were held twice to facilitate discussion to come to a mutual agreement. The involvement of two external reviewers from outside the consortium and two specialists in other cardiovascular fields provided novel feedback. Moreover, timepoints as well as variables should be clearly defined to structure the data dictionaries and standardize data collection at pre-defined key intervals.

These data dictionaries remain a reflection of the KOLs that participated in the consensus process and their own respective opinions about which variables, chosen from society guidelines and existing reporting standards, were regarded as important. This is an issue in every consensus process—a balance should be found between including a sufficiently large and representative group of stakeholders and maintaining a number of participants that facilitate open and effective discussion. In this consensus process, representation from seven countries was achieved with KOLs with an average of 12 years of experience in their field of expertise. Furthermore, in the consensus meetings, eight KOLs participated.

Since the primary use for this data dictionary is the collection of data in the context of the retrospective VASCUL-AID patient cohort studies, retrieval of variables from EHRs for this purpose was considered throughout the consensus process. Despite being comprehensive, encompassing clinical as well as social and surgical information, some variables may be unaccounted for. Clinical research is subject to progressive insights, which may reveal additional clinically relevant risk factors for AAA and PAD progression. These data dictionaries should therefore be regularly updated and improved every 5 years to accommodate these new insights. In addition, these data dictionaries may be employed as a basis of data collection in prospective studies, especially if complemented with new and/or additional variables considered relevant.

This is a comprehensive list of variables to be collected for use in AI applications, but which may also be consulted for health registries and may be employed in clinical as well as AI research in which EHRs are consulted. Selecting variables for datasets used for AI training, testing, and validation only based on existing literature and by consultation of experts could lead to bias, while selection of the most relevant and highly predictive variables generally results in an improved model. Thus, for more comprehensive input variable selection for model training, testing, and validation, most predictive variables from the available data dictionary should be extracted using techniques such as feature engineering and data mining.^51^ The data dictionaries serve as a guide for data collection, recognizing that the feasibility of collecting all variables will vary across EHR systems and may depend on local infrastructure and data quality. Given this variability in EHR completeness for different centres, missing data might be present, and strategies such as variable exclusion or imputation may be necessary to handle these missing variables. To handle missing data, first, it should be explored whether the data are missing completely at random (MCAR) or missing at random (MAR). Subsequently, the data can be imputed using a suitable imputation method specified for the appropriate data type (numerical/categorical). However, in many clinical centres, it is possible to automatically extract data from EHRs. Through automatic extraction, medical files can be searched more comprehensively, resulting in a more complete dataset.

This data dictionary was developed with a specific subset of patients with a retrospective data collection context in mind, in which imaging variables were excluded with intent because they would be analysed separately in our VASCUL-AID imaging studies. However, imaging is an essential cornerstone in AAA and PAD diagnosis, disease management, and treatment and should therefore be included in any research on these diseases as well. These factors have influenced the extent to which these data dictionaries can be applied in other types of research or healthcare domains. The included clinical variables may serve as guidance but should not restrict the number of variables studied in AI research, as these data dictionaries are advised to be updated every 5 years. Moreover, in our prospective study, additional variables that may be prospectively collected will be studied. To increase external validity, multidisciplinary collaboration should be extended to include more vascular specialists and/or general practitioners (in addition to the vascular surgeons, cardiologist, and internal medicine specialist currently involved) and also to include more international collaborators. This can be effectuated by appointing a local coordinator in each participating centre who is responsible for recruitment of additional specialists, both in academic and university hospitals as in regional centres. Furthermore, international collaboration will be sought with international centres outside of Europe in addition to seeking collaboration with different specialism’s societies (e.g. European Society of Cardiology and Cardiovascular and Interventional Radiological Society of Europe). Furthermore, the dependence on retrospective datasets means that not all data are structured, straightforward, and reliable, as EHRs often contain conflicting statements. By the development of a structured data dictionary including definitions for each variable and variable options, we hope to minimize these limitations for EHR data collection.

Another limitation of these data dictionaries is the exclusion of PROs. This is due to their application in retrospective cohort studies, in which PROs are generally not available. For prospective studies, a Delphi consensus has previously identified PROs to be collected for patients with claudication.^23^ In addition, no patients were involved in the creation of these data dictionaries, despite the merits of including patients in consensus processes to ensure its clinical relevance, because adequate insight into (potential) risk factors for AAA and PAD disease progression was required.^52,53^ Instead, core outcomes in both AAA and PAD are currently being investigated with the aim of both including both PROs and involving patients in the consensus process.^54^ These will be applied in the VASCUL-AID cohort studies as well and will be recommended for use in all clinical research on PAD and AAA. Many clinical variables were identified in analysis of focus groups that were conducted for these core outcome sets. For example, patients with PAD acknowledged the importance of claudication, rest pain, and wound infection, but also the level of mobility and multidisciplinary healthcare (manuscripts submitted). In addition, patients with AAA indicated they found variables such as AAA rupture, embolization, AAA growth, the need for dialysis, and the length of stay important (manuscripts submitted). The final core outcomes have been obtained through patient and healthcare provider consensus (manuscripts submitted). These outcomes have been defined based on existing guidelines and reporting standards and will be applied in the VASCUL-AID cohort studies as well, in addition to other validated PROs and PROMs from existing guidelines and reporting standards. By including (consensus-based) PROs and PROMs in these prospective studies, clinical relevance of these outcomes is guaranteed, while outcome preference by patients and healthcare providers is also acknowledged.

## Conclusion

We have established the first data dictionaries within the field of vascular surgery for application in the development of AI-driven tools for patients with PAD and AAA while taking into account ethical and legal perspectives for trustworthy AI. These data dictionaries will form the basis of the data collected in our subsequent VASCUL-AID retrospective cohort studies. More importantly, it may provide a basis for other AI-driven tools in vascular surgery research and similarly incite preventive strategies for patients suffering from AAA and PAD.

## Lead author biography


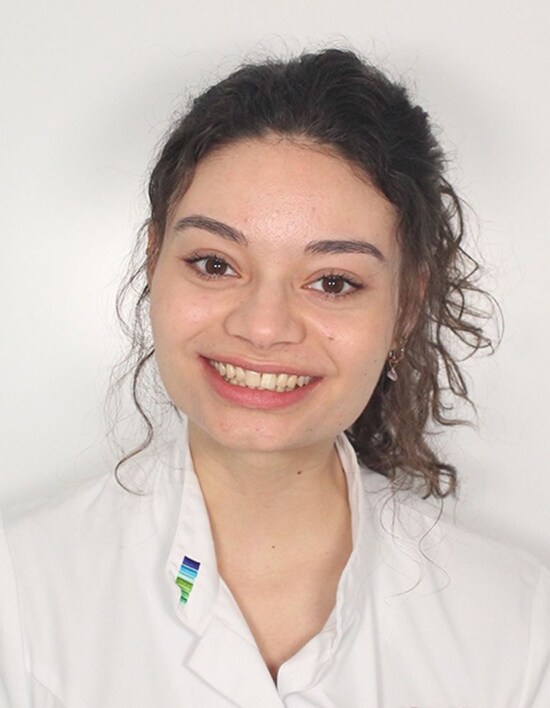


Sabrina L.M. Zwetsloot is a medical doctor and vascular surgery PhD student from the Department of Vascular Surgery at the Amsterdam University Medical Centres, the Netherlands


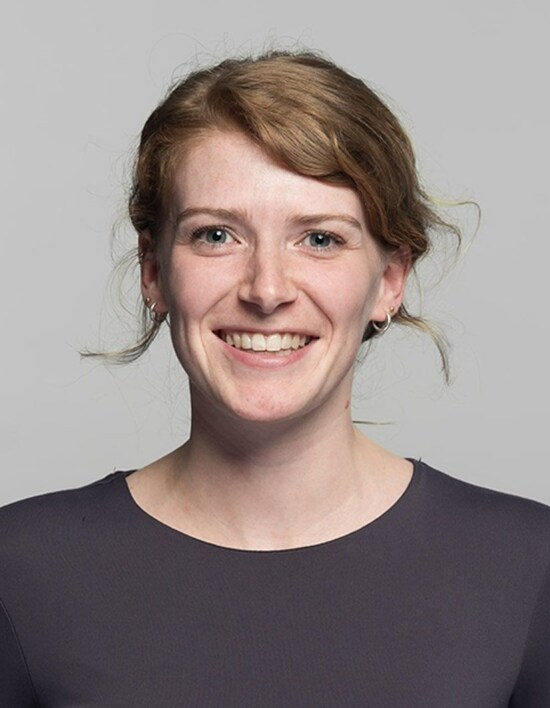


Lotte Rijken is a technical physician and vascular surgery PhD student from the Department of Vascular Surgery at the Amsterdam University Medical Centres, the Netherlands.

## Supplementary Material

ztaf091_Supplementary_Data

## Data Availability

All data are incorporated into the article and its online supplementary material.

## Acknowledgements

VASCUL-AID consortium: Igor Koncar (Medical Faculty University of Belgrade, Belgrade, Serbia; Clinic for Vascular and Endovascular Surgery, Clinical Center of Serbia, Belgrade, Serbia), Ivan Tomic (Medical Faculty University of Belgrade, Belgrade, Serbia; Clinic for Vascular and Endovascular Surgery, Clinical Center of Serbia, Belgrade, Serbia), Marina Dias-Neto (Department of Angiology and Vascular Surgery, Unidade de Saúde Local de São João, Porto, Portugal; RISE-Health, Department of Surgery and Physiology, Faculty of Medicine of the University of Porto, Porto, Portugal*)*, Regent Lee (Nuffield Department of Surgical Sciences, University of Oxford, Oxford, UK), Katarzyna D. Bera (Nuffield Department of Surgical Sciences, University of Oxford, Oxford, UK), Riikka Tulamo (Department of Vascular Surgery, University of Helsinki and Helsinki University Hospital, Helsinki, Finland), Maarit Venermo (Department of Vascular Surgery, University of Helsinki and Helsinki University Hospital, Helsinki, Finland), Mirjami Laivuori (Department of Vascular Surgery, University of Helsinki and Helsinki University Hospital, Helsinki, Finland), Christian-Alexander Behrendt (Department of Vascular and Endovascular Surgery, Asklepios Clinic Wandsbek, Asklepios Medical School, Hamburg, Germany), Stefan P.M. Smorenburg (Amsterdam UMC, location Vrije Universiteit Amsterdam, Vascular Surgery, Amsterdam, the Netherlands; Amsterdam Cardiovascular Sciences, Atherosclerosis & Aortic Diseases, Amsterdam, The Netherlands), Corrette Ploem (Department of Ethics, Law and Humanities, Amsterdam University Medical Center, Location University of Amsterdam, Amsterdam, The Netherlands), Roosmarie Jessen (Department of Ethics, Law and Humanities, Amsterdam University Medical Center, Location University of Amsterdam, Amsterdam, The Netherlands), Bert-Jan H. van den Born (Department of Public and Occupational Health & Department of Vascular Medicine, Amsterdam University Medical Center, Location University of Amsterdam, Amsterdam, The Netherlands), Ronak Delewi (Department of Cardiology, Amsterdam University Medical Center, Location University of Amsterdam, Amsterdam, The Netherlands; Amsterdam Cardiovascular Sciences, Amsterdam, the Netherlands), Jelmer Wolterink (Department of Applied Mathematics, Technical Medical Centre, University of Twente, Enschede, the Netherlands), Noeska Smit (Department of Informatics, University of Bergen, Bergen, Norway; MMIV, Department of Radiology, Haukeland University Hospital, Bergen, Norway), Ivana Išgum (Department of Biomedical Engineering and Physics, Amsterdam University Medical Center, Location University of Amsterdam, Amsterdam, The Netherlands; Department of Radiology and Nuclear Medicine, Amsterdam University Medical Center, Location University of Amsterdam, Amsterdam, The Netherlands; Informatics Institute, Faculty of Science, University of Amsterdam, Amsterdam, The Netherlands), Henk A. Marquering (Department of Biomedical Engineering and Physics, Amsterdam University Medical Center, Location University of Amsterdam, Amsterdam, The Netherlands; Department of Radiology and Nuclear Medicine, Amsterdam University Medical Center, Location University of Amsterdam, Amsterdam, The Netherlands), Fabio Catarinella (Chief Medical Information Officer, Brightfish B.V., Hoofddorp, The Netherlands), Fabien Lareyre (Université Côte d'Azur, CNRS, UMR7370, LP2M, Nice, France; Department of Vascular Surgery, Hospital of Antibes Juan-les-Pins, France), Juliette Raffort (Clinical Chemistry Laboratory, University Hospital of Nice, France; Institute 3IA Côte d’Azur, Université Côte d’Azur, France; Université Côte d'Azur, CNRS, UMR7370, LP2M, Nice, France), Maja Živković (Laboratory for Radiobiology and Molecular Genetics, VINCA Institute of Nuclear Sciences—National Institute of the Republic of Serbia, University of Belgrade, Belgrade, Serbia), Tamara Djuric (Laboratory for Radiobiology and Molecular Genetics, VINCA Institute of Nuclear Sciences—National Institute of the Republic of Serbia, University of Belgrade, Belgrade, Serbia), Aleksandra Stankovic (Laboratory for Radiobiology and Molecular Genetics, VINCA Institute of Nuclear Sciences—National Institute of the Republic of Serbia, University of Belgrade, Belgrade, Serbia).

Each individual served as local coordinator from their respective department and/or centre, each participating in the consensus process with their respective expertise, including proofreading of the manuscript.

VASCUL-AID collaborators: Rupert Bauersachs (CCB-Cardioangiologic Center Bethanien, Frankfurt, Germany; Center of Thrombosis and Hemostasis, University of Mainz, Germany), Manar Khashram (Department of Vascular Surgery Waikato Hospital, University of Auckland, New Zealand).

Collaborators on the VASCUL-AID data dictionary project are involved in the consensus processes with their respective expertise and in proofreading the manuscript.
