# Supplementary material for: The development of a data dictionary with clinical variables for artificial intelligence-driven tools in research on abdominal aortic aneurysms and peripheral arterial disease
Source: Eur Heart J Digit Health. 2025 Aug 20;6(6):1104–12. doi: 10.1093/ehjdh/ztaf091 (PMC12629649; doi:10.1093/ehjdh/ztaf091)
Supplement: ztaf091_Supplementary_Data [file ztaf091_supplementary_data.zip › Supplementary_Table_S2_AAA_Data_Dictionary_27-6-25.docx]

**Supplementary Table S2: Abdominal aortic aneurysm data dictionary**

*This data dictionary provides an overview of the clinical parameters to collect retrospectively from medical patient files from patients with an abdominal aorta aneurysm (AAA).*

**Abdominal aortic aneurysm definition**

An abdominal aortic aneurysm (AAA) has a diameter of 30 mm or more measured in any imaging plane and by any imaging modality.^9^ This includes infrarenal (abdominal aneurysm not involving the renal arteries with neck >10mm), juxtarenal (an aneurysm extending up to but not involving the renal arteries, i.e. neck <10mm), pararenal (an aneurysm involving a renal artery), suprarenal/paravisceral/Crawford type IV thoraco-abdominal (an aneurysm that start above the superior mesenteric artery, involving one or both renal arteries, i.e. no neck, and extends along the abdominal aorta) aneurysms.^18,9^

**Data to collect of AAA patients**

Data collection timepoints were defined to give structure to the variable list and to facilitate the process of future data collection. As patient information may change over time, the variable list was divided into two categories. These are; information at time of the defined ‘index (or: first) visit’ should be reported, and changes in these parameters are to be reported at time of the defined ‘follow-up visit(s)’. Moreover, other events and repeated measurement are included. The following data collection timepoints were identified for this data dictionary:

1. ‘Index visit’: All relevant information that is available at the visit from which data collection starts. Visits should be clearly defined before data collection starts. Hence, the time-point of the visits should be standardized (e.g. inception of the electronic data record, first hospital visit, or date of diagnosis), as well as what types of visits will be included (e.g. elective/acute, what departments, in- outpatient clinic visit, etc.). Moreover, it should be defined who reported the data included in the study, e.g. a vascular surgeon, any other vascular specialist (such as qualified resident, registered nurse or technician), or a healthcare worker in case of an acute setting involved in the treatment.
2. ‘Follow-up visit(s)’: All relevant information that is available from any consecutive follow-up visit after the index visit. Again, it should be clearly defined what follow-up visits should be included for data collection.
3. Other events: All relevant information, more specified information on AAA rupture, AAA interventions, and other performed surgical interventions.
4. Repeated measurements: Cardiovascular imaging, diagnostic tests, laboratory data, and medication records.

**Overview of AAA dictionary categories**

| (1) Relevant data from (baseline) index visit |
| --- |
| **PATIENT STUDY INFORMATION** |
| **MEDICAL REPORT BASELINE DATA** |
| A1: Demographics |
| A2: AAA-related information |
| A3: Other vascular history |
| A4: Cardiac history |
| A5: Comorbidities |
| A6: Social/economic status |
| A7: Substance use |
| A8: General health |
| A9: Family history |
| A10: Vital parameters |
| A11: Imaging data |
| A12: Diagnostic tests |
| A13: Blood test results |
| A14: Microbiology test results |
| A15: Medication |
| A16: Included in other AAA trials/registries/biobank |
| Other remarks |
| (2) Relevant data from follow up visit(s) |
| **FOR EACH FOLLOW-UP VISIT** |
| New vascular report? AAA-related information changed? |
| Did the patient die? |
| Change/new data on: Other vascular history, Cardiac history, Comorbidities, Social/economic status, Substance use, General health, Family history, Vital parameters, Blood test results, Microbiology test results, Medication, Diagnostic tests, Imaging data, Included in other AAA trials/registries/biobank, Other? |
| (3) Other |
| **AAA RUPTURE** |
| Rupture and symptomatic AAA information |
| **AAA INTERVENTION** |
| Per & Post-operative information |
| **OTHER SURGICAL INTERVENTIONS** |
| (4) Repeated data |
| **IMAGING DATA** |
| **DIAGNOSTIC TESTS** |
| ABI & EGC |
| **LABORATORY AND MICROBIOLOGY TESTS** |
| Blood tests & Microbiology tests |
| **MEDICATION RECORDS** |

**AAA data dictionary with all clinical parameters**

| **(1) Relevant data from (baseline) index visit** | | | |  | Relevant information that is available at time of the defined ‘index visit’. |
| --- | --- | --- | --- | --- | --- |
| *Category* | | *Parameter* | *To report* | *Help text* | *Rationale* |
| **PATIENT STUDY INFORMATION** | | | |  |  |
|  | Other general comments about (inclusion of) this patient (optional) | |  |  |  |
| **MEDICAL REPORT BASELINE DATA** | | | |  |  |
| **A1: Demographics** | | |  |  |  |
|  | Date of ‘index visit’ | | dd-mm-yyyy | Index visit as defined in 'information about data collection'. |  |
|  | Sex | | Male, Female |  | Sex (not gender) is a variable relevant to sex-related co-factors and disease progression. |
|  | Birth month | | Months | Month in numbers 1 - 12 | Birth month and year to calculate age at different relevant points in patient trajectory. |
|  | Birth year | | yyyy |  | Birth month and year to calculate age at different relevant points in patient trajectory. |
|  | Ethnicity | | Caucausian, South Asian (India, Pakistan, Bangladesh, Buthan, Nepal), East Asian (Lao, Myanmar, Vietnam) Indonesian, Chinese, Japanese, Asian other (Kazakhstan, Afghanistan, Mongolia, etc), Turkish, Middle Eastern (Saudi Arabia, Jordan, Yemen, Oman, VAE, Palestine, etc), Jewish (Ashkenazi of Sephardi), North African (Algerian, Moroccan, Egyptian, Tunisian, Libyan), Native Australian + Pacific Islanders  Hispanic/Latino, Sub-Saharan African or African-American, Multiple ethnicities, Other, Unknown  > If ‘Multiple ethnicities’ and 'Other': Specify ethnicity | Ethnicity refers to the identification of a group based on a perceived cultural distinctiveness that makes the group into a “people.”^41^  Multiple ethnicity is defined as each parent having a different ethnicity. If multiple, check ethnicities of patients' parents. | Ethnicity refers to the identification of a group based on a perceived cultural distinctiveness that makes the group into a “people.”^41^ Ethnic minority groups have a less favorable disease risk profile than the host population (in this cohort, the host population is the European one).^29^ These ethnicities are the most common in Europe and the specific subclassification were defined with an expert opinion leader. Also, we chose these ethnicities to be able to stratify according to ethnicity, because of genetic differences in disease development.^31^  Multiple ethnicity is defined as each parent having a different ethnicity. |
|  | Country of birth | | Choose from list of all country options, Unknown |  | To be able to distinguish between cultural indicators for AAA. |
|  | Current country of residence | | Finland, Germany, Netherlands, Portugal, Serbia, United Kingdom, Other, Unknown  > If 'Other’: Specify current country of residence |  | To be able to distinguish between cultural indicators for AAA. |
| **A2: AAA-related information** | | | |  | AAA-related information such as characteristics of the AAA, stage of the AAA. |
|  | Year of AAA diagnosis | | yyyy |  | Baseline moment from which to measure consecutive values. Only ‘year’ is chosen as the exact date might not be known and to prevent a surplus of missing values. |
|  | Reason for index visit with vascular surgeon | | Newly-diagnosed, Acute admission, Pre-operative surveillance, First post-operative control, Post-operative surveillance, Planned admission related to AAA intervention, Referral from another hospital, Visit to the Emergency Department (ED) (but no admission), Other, Unknown  > If 'Other’: Specify other reason for index visit with vascular surgeon  > If ‘Planned admission related to AAA intervention’: Specify AAA INTERVENTION parameters | -Newly-diagnosed: specifically first ever visit to the vascular surgeon with a new diagnosis of AAA -Acute admission: admission related to AAA was unforeseen. A patient could have been admitted from the emergency department, the outpatient clinic, or from home.  -Surveillance (pre-op): any kind of routine visit to evaluate symptoms, disease progression or to carry out routine measurements (e.g. lab tests or imaging) before any intervention or operation has been performed.  -First postoperative control: the first postoperative follow-up visits within 3 months* after each intervention or operation.  -Post-operative surveillance: visits after the 3 month postoperative period has surpassed and routine visits are performed.  -Planned admission related to AAA intervention: this includes all interventions performed by a vascular surgeon/resident and all interventions performed at request of the vascular surgeon by an interventional radiologist or surgeon of another specialty that is performed in the hospital. This also includes a planned visit of under 24h in which a planned AAA intervention will be performed.  -Referral from another hospital: when the patient is referred from another specialist in another hospital for their disease. -Visit to the Emergency Department (ED), but no hospital admission. | To establish a baseline ‘reason for visit’, from which to track disease progression.  * arbitrarily chosen because most follow-up visits take place within 3 months of the intervention. |
|  | > If ‘Acute admission’: Specify reason for acute admission | | Symptomatic AAA, (contained) Ruptured AAA, Complications related to previous AAA intervention, Treatment for AAA-related infection, Other, Unknown  > If 'Other’: Specify other reason for acute admission  > If ‘Symptomatic AAA’ or ‘(contained) ruptured AAA’: Specify AAA RUPTURE parameters  > If ‘Treatment for AAA-related infection’ or ‘Complications related to previous AAA intervention’, if applicable: Specify AAA INTERVENTION parameters | -Symptomatic AAAs are those presenting with abdominal and/or back pain, tender AAA at palpation, or embolic events, but without breach of the aortic wall.^9^ A symptomatic AAA is an indication of progression of disease and have a higher rupture risk than asymptomatic aneurysms. Progression to a symptomatic aneurysm and/or rupture may be investigated in these patients. -rAAA is defined as an acute haemorrhage from the AAA outside the true aortic wall with the presence of retroperitoneal and/or intraperitoneal blood.^9^ Rupture indicates aneurysm progression and is a core outcome of AAA. |  |
|  | > If ‘Inpatient admission’: Complications during in-hospital period? | | Yes, No, Unknown |  |  |
|  | >> If ‘Yes’: Specify complications during in-hospital period and Clavien-Dindo classification (see classification) (Only fill in the amount of complications that were there, the other fields can be left empty) | | Fill in for each complication:   - Complications during in-hospital period: Congestive heart failure (decompensatio cordis), Acute heart failure, Cardiac arrhythmia, Myocardial infarction, Atrial fibrillation (AF), Urinary tract infection (UTI), Renal function deterioration, Fever without known cause, Pneumonia, COPD exacerbation, Stroke, TIA, Ileus, Complications related to (endo)graft/bypass, Other, Unknown   > If ‘Other’: Specify other complication   - Clavien-Dindo classification: Grade I, Grade II, Grade IIIa, Grade IIIb, Grade IVa, Grade IVb, Grade V   > Specify other comments about complications (optional). If it is about a specific complication, specify the complication. |  |  |
|  | Location of index visit | | Emergency department, Outpatient clinic, Inpatient (clinic), Unknown |  | Index visit as defined. |
|  | Type of abdominal aortic aneurysm | | Infrarenal, Juxtarenal, Pararenal, Suprarenal, Other, Unknown  > If 'Other’: Specify other type of abdominal aortic aneurysm | -Infrarenal (abdominal aneurysm not involving the renal arteries with neck >10mm)  -Juxtarenal (an aneurysm extending up to but not involving the renal arteries, i.e. neck <10mm) -Pararenal (an aneurysm involving a renal artery) -Suprarenal=paravisceral=Crawford type IV thoraco-abdominal (an aneurysm that extends up to the superior mesenteric artery, involving one or both renal arteries, i.e. no neck) aneurysms.  Note: Crawford type I-III thoraco-abdominal aneurysms should be excluded. | We focus solely on abdominal aortic aneurysms, including:   - Infrarenal - Juxtarenal - Pararenal - Suprarenal=paravisceral=Crawford type IV thoraco-abdominal   Localization of the AAA determines treatment strategy. |
|  | Maximum diameter AAA (mm) | | Diameter in millimeters | Reported at time of index visit with vascular surgeon. If measured on two modalities for the index visit; report the measurement on CT(A), second best: US, or after, any other modality. The most recently measured maximum diameter should be reported.  If someone already has had an AAA intervention, this question does not have to be answered.   If unknown leave it empty. | AAA diameter determines indication for surgery. Through this variable a baseline AAA diameter is provided and we are able to track its growth.^9^  Select the imaging modality that has been used and note the diameter of the aneurysm.  If someone already has had an AAA intervention, this question does not have to be answered. |
|  | > Imaging modality of the maximum AAA diameter measurement | | US, CT, CTA (CT Angiography), MRI, MRA (MR Angiography), PET-CT, Other, Unknown  > If 'Other’: Specify other imaging modality |  | Imaging modalities differ in their accuracy, moreover there is a considerable inter-observer variability for some (namely, US). It is important to note imaging modality so this can be considered in assessment of the diameter. The measured aortic diameter depends on the method used.^9^ |
|  | > Measurement method maximum diameter | | Antero-posterior (AP), CLL (central lumen line) or MPR (multiplanar reconstruction), Other, Unknown  > If 'Other’: Specify other measurement method |  | The diameter measurement method should be known to make a fair comparison between diameters measured at different time points and to compare the diameter between patients. ESVS AAA guidelines 2019: ‘the measured aortic diameter significantly depends on the method used’. |
|  | Aneurysm shape | | Fusiform, Saccular, Unknown | -Fusiform AAA was defined as aneurysm involving the whole circumference of the aortic wall. -Saccular AAA is as a spherical aneurysm involving only a portion of the aortic circumference.^31^ | The shape of the AAA may influence the progression rate of the aneurysm.^9^ |
|  | Penetrating Aortic Ulcer (PAU) | | Yes, No, Unknown  > If ‘Yes’: Specify year of diagnosis penetrating aortic ulcer (PAU) | A PAU is defined as ulceration of an atherosclerotic plaque that penetrates through the aortic intima resulting in a variable amount of haematoma within the aortic wall.^9^  If year is unknown, leave empty. |  |
|  | > If ‘Yes’: Specify  Penetrating aortic ulcer (PAU) location | | Ascending aorta, Arch, Descending aorta, Abdominal aorta, Other, Unknown  > If 'Other’: Specify other location PAU |  |  |
|  | Pseudoaneurysm/false aneurysm | | Yes, No, Unknown  > If ‘Yes’: Specify year of diagnosis Pseudoaneurysm/false aneurysm | Pseudoaneurysm is defined as dilatation of the aorta due to disruption of all wall layers, which is only contained by peri-aortic connective tissue.^9^  If year is unknown, leave empty. | Progression of PAU, or other arterial wall injury may lead to an pseudoaneurysm formation.^9^ |
|  | Inflammatory aneurysm | | Yes, No, Unknown  > If ‘Yes’: Specify year of diagnosis inflammatory aneurysm | An inflammatory AAA is defined by (1) an unusually thickened aneurysm wall, (2) shiny white peri-aneurysmal and retroperitoneal fibrosis, and (3) dense adhesions of adjacent intra-abdominal structures.  If year is unknown, leave empty. | Inflammation of the AAA may influence the progression rate of the aneurysm.^9^ |
|  | Mycotic aneurysm | | Yes, No, Unknown  > If ‘Yes’: Specify year of diagnosis mycotic aneurysm | Mycotic or primary infected aortic aneurysms are caused by septic emboli to the vasa vasorum, leading to an infectious degeneration of the arterial wall and aneurysm formation. The diagnosis of MAA is based on a combination of: (1) clinical presentation: abdominal/back pain, fever, sepsis/shock. (2) laboratory tests: C-reactive protein increase, Leucocytes increase, positive blood culture or aortic tissue culture. (3) CT findings: saccular/multi-lobular/eccentric, Peri-aortic gas/soft tissue mass, rapid expansion (days) and/or rupture, Atypical location (eg para-visceral) or multiple aneurysms in different locations.^9^  If year is unknown, leave empty. | An AAA being mycotic may influence the progression rate of such an aneurysm. |
|  | Previous AAA intervention performed in another hospital? | | Yes, No, Unknown  > If yes: Specify AAA INTERVENTION parameters | Previous intervention before index visit with the vascular surgeon in another hospital. | The need for previous interventions gives an indication of disease severity in the past and current disease progression. |
|  | > If ‘Yes’: Were there any AAA intervention-related complications? | | No, Yes general AAA intervention-related complications, Yes endograft-related complications, Unknown |  |  |
|  | >> If ‘Yes general AAA intervention-related complications‘: Specify general AAA intervention-related complication(s) and Clavien-Dindo classification (see classification below) (Only fill in the amount of complications that were there, the other fields can be left empty) | | Fill in for each complication:   - Complications intervention-related: Bleeding, Thrombosis, Peripheral embolization, Access site hematoma, Access site seroma (lymphocele), Access site wound infection superficial, Access site wound infection deep, Arterial perforation or rupture, Artery dissection, Renal impairment, Dialysis, Stroke, TIA, Ileus, Gastroparesis, Bowel ischemia, Spinal cord ischemia, Erectile dysfunction, Other, Unknown   > If ‘Other’: Specify other complication   - Clavien-Dindo classification: Grade I, Grade II, Grade IIIa, Grade IIIb, Grade IVa, Grade IVb, Grade V   > Specify other comments about complications (optional). If it is about a specific complication, specify the complication. |  |  |
|  | >> If ‘Yes endograft-related complications‘: Specify endograft-related complication(s) and Clavien-Dindo classification (see classification below) (Only fill in the amount of complications that were there, the other fields can be left empty) | | Fill in for each complication:   - Complications endograft-related: (Endo)graft infection, (Endo)graft occlusion, (Endo)graft migration, Device erosion through aortic or iliac wall, Buttock/leg claudication/ischemia, Endoleak, Other, Unknown   > If ‘Other’: Specify other complication   - Clavien-Dindo classification: Grade I, Grade II, Grade IIIa, Grade IIIb, Grade IVa, Grade IVb, Grade V   > Specify other comments about complications (optional). If it is about a specific complication, specify the complication. |  |  |
|  | >> If ‘Yes endograft-related complications‘:  If applicable: Specify the type of endoleak | | No, Type I, Type Ia, Type Ib, Type II, Type III, Type IV, Type V, Unknown | If not applicable, leave empty. |  |
|  | Previous AAA intervention performed in current hospital? | | Yes, No, Unknown  > If ‘Yes’: specify AAA INTERVENTION parameters | Previous intervention before index visit with the vascular surgeon in the current hospital. | The need for previous interventions gives an indication of disease severity in the past and current disease progression. |
|  | > If ‘No’ previous intervention: Specify reason no intervention | | Below threshold for elective AAA repair, Patient declined surgery, Risk of intervention is too high for patient, Above threshold for elective AAA repair and determining treatment plan, Other, Unknown  > If 'Other’: Specify other reason no intervention | -Below threshold for elective AAA repair (No indication): The treating physician decided that there was no indication for operative treatment, e.g. because disease progression was not severe enough to warrant operative treatment.  -Patient declined intervention: patient chose to not be operated. -Risk of intervention too high for patient: the risk of intervention was too high because of comorbidities, age, or overall health status of patient. This may be decided by physician or be a decision made through shared-decision making.  -Above threshold for elective AAA repair (indication for intervention) and in the process of determining treatment plan. | To evaluate what factors are important in decision-making to undergo an intervention for AAA. |
| **A3: Other vascular history** | | | |  | For this subset of questions we want to identify how much some frequent vascular diseases contribute to AAA disease progression or cardiovascular adverse events. When a patient has multiple vascular comorbidities, this could be an indication of extensive vascular disease.  For the vascular history, we tried to include as many comorbidities that are either 1) already known to have an association with AAA or 2) are common, so that subgroup analysis or stratification may be applied for potential confounders. |
|  | Peripheral artery disease (PAD) of lower limbs | | Yes, No, Unknown  > If ‘Yes’: Specify year of diagnosis Peripheral Artery Disease (PAD) of lower limbs | Lower extremity PAD: atherosclerotic obstruction from the aorto-iliac segments to the pedal arteries.^2^  If year is unknown, leave empty. | PAD is associated with abdominal aneurysms.^32^ |
|  | > If ‘Yes’: PAD Fontaine classification | | Fontaine stage I: asymptomatic,  Fontaine stage II: intermittent claudication,  Fontaine stage IIa: intermittent claudication walking >200m,  Fontaine stage IIb: intermittent claudication walking <200m,  Fontaine stage III: rest pain,  Fontaine stage IV: Ulceration or gangrene, Unknown | The Fontaine classification is a classification systems for peripheral arterial disease. | Fontaine and Rutherford classification are evidence-based classification systems for peripheral arterial disease and indicate disease severity. To be able to have an objective measure to track PAD disease progression. |
|  | > If ‘Yes’: Rutherford classification | | Grade 0 Category 0: Asymptomatic, Grade I Category 1: Mild claudication, Grade I Category 2: Moderate claudication,  Grade I Category 3: Severe claudication,  Grade II Category 4: Ischemic rest pain,  Grade III Category 5: Minor tissue loss, Grade III Category 6: Major tissue loss,  Unknown | The Rutherford classification is a classification systems for peripheral arterial disease. - Grade 0, Category 0: asymptomatic – no hemodynamically significant occlusive disease  - Grade I, Category 1: mild claudication  - Grade I, Category 2: moderate claudication  - Grade I, Category 3: severe claudication - Grade II, Category 4: ischaemic rest pain - Grade III, Category 5: minor tissue loss – non-healing ulcer, focal gangrene with diffuse pedal ischemia - Grade III, Category 6: major tissue loss – extending above transmetatarsal level, functional foot no longer salvageable | Fontaine and Rutherford classification are evidence-based classification systems for peripheral arterial disease and indicate disease severity. To be able to have an objective measure to track PAD disease progression. |
|  | > If ‘Yes’: Intervention performed for PAD? | | Yes, No, Unknown  > If ‘Yes’: Specify OTHER SURGICAL INTERVENTION parameters |  |  |
|  | Previous Acute limb ischemia | | Yes, No, Unknown  > If ‘Yes’: Specify date of diagnosis Acute Limb Ischemia | ALI is characterized by a sudden decrease in arterial perfusion of the limb, with a potential threat to the survival of the limb, requiring urgent evaluation and management. ALI is considered when the symptom duration is less than two weeks.^33^  If date is unknown, leave empty. |  |
|  | Aortic dissection? | | Yes asymptomatic acute, Yes asymptomatic chronic, Yes symptomatic acute, Yes symptomatic chronic, Yes type unknown, No, Unknown  > If ‘Yes’: Specify date of diagnosis aortic dissection | ● Definition of acute aortic dissection: Aortic dissection is defined as disruption of the medial layer provoked by intramural bleeding, resulting in separation of the aortic wall layers and subsequent formation of a TL and an FL with or without communication.  ○Acute asymptomatic: without clinical symptoms ○Acute symptomatic: with clinical symptoms such as chest pain, back pain, pleural effusion, etc. ● Chronic dissection: >90 days. ○Chronic asymptomatic: no symptoms. ○Chronic symptomatic: signs of (contained) rupture with complaints as mentioned under acute symptomatic.^33^  If date is unknown, leave empty. | Aortic dissection: a tear in the intimal layer and subsequent blood flow through the tear into the media creating a false lumen.^9^  A distinction is made between acute and chronic dissection because these may have different etiologies and different outcomes that may be related to AAA.^34^ |
|  | > If ‘Yes’: Type of Aortic dissection | | Type A, Type B, Unknown | Type A involves the ascending aorta, regardless of the site of the primary intimal tear. Type A dissection is defined as a dissection proximal to the brachiocephalic artery. Type B aortic dissection originating distal to the left subclavian artery and involving only descending aorta. |  |
|  | > If ‘Yes’: Intervention performed for aortic dissection? | | Yes, No, Unknown  > If ‘Yes’: Specify OTHER SURGICAL INTERVENTION parameters |  | The need for an intervention gives an indication of disease severity. |
|  | Iliac artery aneurysm (IAA)? | | Yes, No, Unknown | IAA definition: Dilatation of the vessel to more than 1.5 times its normal diameter.^9^ |  |
|  | > If ‘Yes’: Location of iliac aneurysm | | A. iliaca communis – Right, A. iliaca communis – Left, A. iliaca externa – Right, A. iliaca externa – Left, A. iliaca interna – Right, A. iliaca interna – Left, Unknown | Multiple options possible. | To localize the IAA, to make sure the same IAA can be followed over time. |
|  | > If ‘Yes’:  Maximum iliac aneurysm diameter (largest IAA aneurysm diameter) (mm) | | Diameter in millimeters for each IAA separately | Reported at time of index visit with vascular surgeon. If measured on two modalities for the index visit; report the measurement on CT(A), second best: US, or after, any other modality. The most recently measured maximum diameter should be reported.  If someone already has had an AAA intervention, this question does not have to be answered.   If unknown, leave empty. | IAA diameter determines indication for surgery.^9^ This variable provides a baseline IAA diameter and the ability to track growth. |
|  | >> If ‘Max diameter reported’: Imaging modality of maximum iliac aneurysm diameter measurement | | US, CT, CTA (CT angiography), MRI, MRA (MR angiography), PET-CT, Other, Unknown  > If 'Other’: Specify other imaging modality |  | The measured aortic diameter depends on the method used.^9^ Imaging modalities differ in their accuracy, moreover there is a considerable inter-observer variability for some (namely, US). It is important to note imaging modality so this can be considered in assessment of the diameter. |
|  | >> If ‘Max diameter reported’:  Measurement method of maximum iliac aneurysm diameter | | Anteroposterior (AP), CLL (central lumen line) or MPR (multiplanar reconstruction), Other, Unknown  > If 'Other’: Specify other measurement method |  | The measured aortic diameter depends on the method used.^9^ The diameter measurement method should be known to make a fair comparison between diameters measured at different time points and to compare the diameter between patients. |
|  | Thoracic aortic aneurysm | | Yes, No, Unknown  > If ‘Yes’: Specify year of diagnosis thoracic aortic aneurysm |  |  |
|  | > If ‘Yes’: Specify type of Thoracic aortic aneurysm | | Ascending, Arch, Descending, Other, Unknown |  |  |
|  | Intracranial aneurysm | | Yes, No, Unknown  > If ‘Yes’: Specify year of diagnosis Intracranial aneurysm |  |  |
|  | Popliteal aneurysm | | Yes, No, Unknown  > If ‘Yes’: Specify year of diagnosis Popliteal aneurysm |  |  |
|  | Other aneurysm | | Yes, No, Unknown  > If ‘Yes’: Specify year of diagnosis other aneurysm |  |  |
|  | > If ‘Yes’: Specify location of other aneurysm | |  |  |  |
|  | Carotid stenosis? | | No, Yes left, Yes right, Yes left and right, Unknown |  |  |
|  | Intervention performed for carotid stenosis? | | Yes, No, Unknown  > If ‘Yes’: Specify OTHER SURGICAL INTERVENTION parameters |  | The need for an intervention gives an indication of disease severity. |
|  | Other occlusions elsewhere? | | Yes, No, Unknown |  |  |
|  | > If ‘Yes’: Report which artery/arteries are occluded | |  |  |  |
|  | Genetic connective tissue disorder? | | No, Yes Ehler-Danlos, Yes Marfan, Yes Loeys-Dietz, Yes other, Unknown  > If ‘Yes other’: Specify other genetic connective tissue disorder  > If not ‘No’: Specify year of diagnosis genetic vascular/connective tissue disorder | If year is unknown, leave empty. | Genetic vascular/connective tissue disorders can result in a different disease progression of the aneurysm compared to an AAA without a disorder. |
|  | > If ‘Yes Ehler-Danlos’: Specify Ehler-Danlos Syndrome (EDS) type | | Classical EDS, Vascular EDS (type IV), Hypermobile EDS (type III), Other, Unknown  > If ‘Other’: Specify other Ehler-Danlos Syndrome (EDS) type |  | There are several Ehler-Danlos syndrome types and some have more vascular impact than others. |
|  | > If ‘Yes Loeys-Dietz’: Specify Loeys-Dietz Syndrome (LDS) type | | Type 1 (LDS-1), Type 2 (LDS-2), Other, Unknown  > If ‘Other’: Specify other Loeys-Dietz Syndrome (LDS) type |  |  |
|  | Other vascular diseases/pathologies | | Yes, No, Unknown  > If ‘Yes’: Specify other vascular disease(s)/pathology, and specify  year of diagnosis other vascular disease(s)/pathology | If year is unknown, leave empty. | Relevant vascular diseases/conditions for which people have been hospitalized or require medication. Or, diseases that you deem relevant. |
| **A4: Cardiac history** | | |  |  | For this subset of questions we want to identify the cardiac medical history including all adverse cardiac events, to evaluate the cardiovascular status of the patient. Besides, cardiac complications are estimated to cause more than 40% of peri-operative deaths after non-cardiac surgery and the level of cardiac risk should therefore be assessed.^9^  For the cardiac history, we tried to include as many comorbidities that are either 1) already known to have an association with AAA or 2) are common, so that subgroup analysis or stratification may be applied for potential confounders. To make the questions as complete and standardized as possible, we have used the standard reporting outcomes of Oderich et al. (for AAA patients), and integrated these with reporting standards of Behrendt et al. (for PAD patients).^18,22^ |
|  | Cardiac arrhythmia | | Yes, No, Unknown |  |  |
|  | > If ‘Yes’: Specify Cardiac arrhythmia: former or current | | Former, Current, Unknown |  |  |
|  | > If ‘Yes’: Specify  type cardiac arrhythmia | | Atrial Fibrillation, Atrial Flutter, Other, Unknown  > If ‘Other’: Specify other type of cardiac arrhythmia | If ‘Other’ type is unknown, leave empty. |  |
|  | > If ‘Yes’: Does patient have a pacemaker? | | Yes, No, Unknown |  |  |
|  | > If ‘Yes’: Intervention performed for cardiac arrhythmia? | | Yes, No, Unknown  > If ‘Yes’: Specify OTHER SURGICAL INTERVENTION parameters |  |  |
|  | CHADSVASC score | | 0, 1, 2, 3, 4, 5, 6, 7, 8, 9, Unknown | If reported by the physician. |  |
|  | Congestive Heart Failure (decompensatio cordis) | | Yes, No, Unknown  > If ‘Yes’: Specify year of diagnosis |  |  |
|  | > If ‘Yes’: Congestive Heart Failure (decompensatio cordis): former or current | | Former, Current |  |  |
|  | > If ‘Current’: New York Heart Association (NYHA)-Classification (I-IV) for heart failure | | I No limitation of physical activity,  II Slight limitation of physical activity,  III Marked limitation of physical activity,  IV Symptoms (fatigue, palpitation, shortness of breath or chest pain) of heart failure at rest, Unknown |  |  |
|  | > If ‘Yes’: Specify ejection fraction | | >45%, 25% - 45%, <25%, Unknown |  |  |
|  | Valvular heart disease | | Yes, No, Unknown  > If ‘Yes’: Specify year of diagnosis | If year is unknown, leave empty. |  |
|  | > If ‘Yes’: Specify affected valve | | Aortic, Mitral, Pulmonary, Tricuspid, Unknown |  |  |
|  | > If ‘Yes’: Specify valvular heart disease type | | Regurgitation, Stenosis, Atresia, Other, Unknown  > If ‘Other’: Specify other type of valvular heart disease |  |  |
|  | Coronary artery disease/Angina pectoris | | Yes, No, Unknown  > If ‘Yes’: Specify year of diagnosis | If year is unknown, leave empty. |  |
|  | > If ‘Yes’: Coronary artery disease/Angina Pectoris: Former or Current | | Former, Current |  |  |
|  | > If ‘Yes’: Unstable Angina? | | Yes, No, Unknown |  | Unstable angina is defined as myocardial ischaemia at rest or on minimal exertion in the absence of acute cardio myocyte injury/necrosis.^35^ |
|  | > If ‘Yes’:  Intervention performed for Coronary artery disease? | | Yes, No, Unknown  > If ‘Yes’: Specify OTHER SURGICAL INTERVENTION parameters |  |  |
|  | (Prior) Myocardial Infarction | | Yes, No, Unknown  > If ‘Yes’: Specify year of diagnosis | If year is unknown, leave empty.  If multiple Myocardial Infarctions, report each occurrence as below: * yyyy  * yyyy  If multiple myocardial infarctions in the same year report the same year twice. |  |
|  | > If ‘Yes’: Specify type of infarction | | STEMI, NSTEMI, Unknown |  |  |
|  | > If ‘Yes’: Intervention performed for (prior) Myocardial Infarction? | | Yes, No, Unknown  > If ‘Yes’: Specify OTHER SURGICAL INTERVENTION parameters |  |  |
|  | Other cardiac pathology | | Yes, No, Unknown  > If ‘Yes’: Specify other cardiac pathology, and specify year of diagnosis other cardiac pathology | If year is unknown, leave empty. | Relevant cardiac diseases/conditions for which people have been hospitalized or require medication. Or, diseases that you deem relevant. |
| **A5: Comorbidities** | | |  |  | For this subset of questions we want to identify other comorbidities to analyze potential risk factors, and to assess patients’ overall health status. For example people with lung disease or kidney problems are more likely to suffer complications after vascular surgery than those without.^9^  For comorbidities, we tried to include as many comorbidities that are either 1) already known to have an association with AAA or 2) are common, so that subgroup analysis or stratification may be applied for potential confounders. To make the questions as complete and standardized as possible, we have used the standard reporting outcomes of Oderich et al. (for AAA patients), and integrated these with reporting standards of Behrendt et al. (for PAD patients). Some parameters are added by the research group to encompass all major comorbidities.^18,22^ |
|  | Hypertension | | Yes, No, Unknown  If ‘Yes’: Specify year of diagnosis | When reported in medical file or when patient uses blood pressure lowering medicine (long term).   Examples of blood lowering medicine: amlodipine, barnidipine, diltiazem, felodipine, lacidipine, lercanidipine, nicardipine, nifedipine, verapamil. |  |
|  | > If ‘Yes’: Blood pressure controlled with medication? | | Yes, No, Unknown | Eg. amlodipine, barnidipine, diltiazem, felodipine, lacidipine, lercanidipine, nicardipine, nifedipine, verapamil |  |
|  | Chronic Kidney disease | | Yes, No, Unknown  > If ‘Yes’: Specify year of diagnosis | If year is unknown, leave empty. | KDIGO classification.^36^ |
|  | Dialysis dependence | | Yes, No, Unknown  > If ‘Yes’: Specify start year of dialysis | If start year is unknown, leave empty. |  |
|  | Pulmonary disease? | | Yes, No, Unknown |  |  |
|  | > If ‘Yes’: Dyspnea? | | No, Mild (only during physical activity), Severe (in rest and during physical activity), Severe and medically necessary use of supplemental oxygen, Unknown |  |  |
|  | > If ‘Yes’: Asthma? | | Yes, No, Unknown |  |  |
|  | > If ‘Yes’: Pulmonary hypertension? | | Yes, No, Unknown |  |  |
|  | > If ‘Yes’: Idiopathic pulmonary fibrosis (IPF)? | | Yes, No, Unknown  > If ‘Yes’: Specify year of diagnosis | If year is unknown, leave empty. |  |
|  | COPD | | Yes, No, Unknown |  |  |
|  | > If ‘Current’: COPD Gold classification | | GOLD 1 - mild: FEV1 ≥80% predicted,  GOLD 2 - moderate: 50% ≤ FEV1 <80% predicted,  GOLD 3 - severe: 30% ≤ FEV1 <50% predicted,  GOLD 4 - very severe: FEV1 <30% predicted, Unknown |  |  |
|  | > If ‘Current’: Specify treatment | | No Treatment, Medical Treatment, Home Oxygen, Other, Unknown  > If ‘Other’: Specify COPD treatment | Multiple options possible. |  |
|  | Other pulmonary disease? | | Yes, No, Unknown  > If ‘Yes’: Specify other pulmonary disease |  |  |
|  | Ulcus pepticum | | Yes, No, Unknown  > If ‘Yes’: Specify year of diagnosis | If year is unknown, leave empty. |  |
|  | Diabetes | | No, DM type 1, NIDDM (among which DM type 2), Gestational Diabetes, Unknown  > If not ‘No’ or ‘Unknown’: Specify year of diagnosis | DM: diabetes mellitus NIDDM: Non-insulin-dependent diabetes mellitus  If year is unknown, leave empty. |  |
|  | > If ‘NIDDM’: Specify control of NIDDM | | Uncontrolled, Controlled with oral blood glucose lowering medication, Other, Unknown  > If ‘Other’: Specify other control of NIDDM |  |  |
|  | Cerebro Vascular Accident (CVA) | | No, Yes stroke (hemorrhagic), Yes stroke (ischemic), Yes stroke type unspecified, Yes TIA, Yes other, Unknown  > If ‘Other’: Specify other CVA  > If not ‘No’ or ‘Unknown’: Specify year of diagnosis | If year is unknown, leave empty. |  |
|  | Neurological disorders | | No, ALS, Epilepsy, MS, Parkinson, Alzheimer, Dementia, Migraine, Other, Unknown  > If not ‘No’ or ‘Unknown’: Specify year of diagnosis  > If ‘Other’: Specify | If year is unknown, leave empty.  If multiple Neurological disorders, report each disorder as below: * yyyy  * yyyy |  |
|  | (previous) Malignancy | | Yes, No, Unknown  > If ‘Yes’: Specify year of diagnosis | Specify both the malignancy (as specified below) and the year of diagnosis. if multiple, report all.  Write it as this: * Malignancy - yyyy * Malignancy - yyyy  If year is unknown, leave empty. |  |
|  | > If ‘Yes’: Location of primary tumor | | Lungs, Breast, Colorectal, Prostate, Liver, Pancreas, Stomach, Esophagus, Uterus, Thyroid, Melanoma, Skin, Lymphoma, Ovary, Bladder, Bone, Leukemia, Desmoïd tumor, Brain tumor, Kidney, Other, Unknown  > If ‘Other’: Specify location of other primary tumor |  |  |
|  | > If ‘Yes’: Specify TNM-classification | | T(0-4), N(0-2), M(0-1) | Report the T(0-4)N(0-2)M(0-1) classification according this format:  Tumor (exact name as reported above): TscoreNscoreMscore  For example: Lungs: T1N1M0  If unknown, leave empty. |  |
|  | > If ‘Yes’: Treatment for Malignancy | | No treatment performed, Surgery, Chemotherapy, Radiation, Hormone therapy, Hyperthermia, Palliative treatment, Other, Unknown  > If ‘Other’: Specify other treatment for malignancy  > If ‘Surgery’: Specify OTHER SURGICAL INTERVENTION parameters | Multiple choice because combination of treatment is possible. |  |
|  | Thyroid disease? | | No, Yes hypothyroid, Yes hyperthyroid, Unknown  > If not ‘No’ or ‘Unknown’: Specify year of diagnosis | If year is unknown, leave empty. |  |
|  | Hyperlipidemia | | Yes, No, Unknown  > If ‘Yes’: Specify year of diagnosis | When reported in medical file or when patient uses statins.  If year is unknown, leave empty. |  |
|  | > If ‘Yes’: Hyperlipidemia controlled with medication? | | Yes, No, Unknown | E.g. Statins |  |
|  | Liver hepatitis | | Yes, No, Unknown  > If ‘Yes’: Specify year of diagnosis | If year is unknown, leave empty. |  |
|  | Liver cirrhosis | | Yes, No, Unknown  > If ‘Yes’: Specify year of diagnosis | If year is unknown, leave empty. |  |
|  | Other liver disease/pathology? | | Yes, No, Unknown  > If ‘Yes’: Specify other liver disease/pathology  > If ‘Yes’: Specify year of diagnosis | If year is unknown, leave empty. |  |
|  | Gastrointestinal pathology? | | No, Inflammatory bowel disease (Crohn's disease or ulcerative colitis), Gastric ulcer, Barrett Esophagus, History of cholecystitis/cholecystolithiasis, Other, Unknown  > If ‘Other’: Specify other gastrointestinal pathology  > If not ‘No’ or ‘Unknown’: Specify year of diagnosis | If year is unknown, leave empty.  If multiple Gastrointestinal disease, report each disease as below: * yyyy  * yyyy |  |
|  | Rheumatoid arthritis? | | Yes, No, Unknown  > If ‘Yes’: Specify type of Rheumatoid arthritis  > If ‘Yes’: Specify year of diagnosis | If unknown, leave empty.  If year is unknown, leave empty. |  |
|  | HIV or AIDS? | | No, Yes HIV, Yes AIDS, Unknown  > If ‘Yes’: Specify year of diagnosis |  |  |
|  | Clinical diagnosis of depression? | | Yes, No, Unknown |  |  |
|  | Other relevant comorbidities? | | Yes, No, Unknown  > If ‘Yes’: Specify other relevant comorbidities  > If ‘Yes’: Specify year of diagnosis | Specify both the comorbidity and the year of diagnosis (multiple) Write it as this: * Comorbidity - yyyy * Comorbidity - yyyy | Relevant comorbidities: diseases/conditions for which people have been hospitalized or require medication. Or, diseases that you deem relevant. |
|  | Other previous surgery/intervention performed? | | Yes, No, Unknown  > If ‘Yes’: Specify previous surgery/intervention performed in OTHER SURGICAL INTERVENTION parameters  > If ‘Yes’: Specify year of surgery | Include all surgeries that have been performed (that you did not input yet). | Surgery temporarily or permanently alters the physical condition of the body and has a significant impact systemically; therefore, it is important to note whether a patient has had surgery to be able to evaluate or correct for its effect.^37^ To gain an overview of surgical history (also surgery performed in other hospitals), other medical conditions for which the patient received surgery, and the burden of comorbidities. |
| **A6: Social and economic status** | | | |  | Research has proven that socioeconomic status has an impact on cardiovascular outcomes.^38^ |
|  | Partner | | Married or with (registered) partnership, In relationship, Single, Widowed, Divorced, Other, Unknown  > If ‘Other’: Specify other partnership |  |  |
|  | Work (or previous work) | |  |  |  |
|  | Living circumstances | | Home independently, Home with help, Nursery home, Living with relatives, Other, Unknown  > If ‘Other’: Specify other living circumstances | Home with help may be help from government-assisted facilities such as a nurse or help from relatives. | Living at home without the need of additional help indicates that a person is at least self-sufficient in their daily needs. |
|  | Education | | Lower (eg. senior secondary vocational education; practical studies), Intermediate (eg. higher vocational education; combines theoretical and practical studies), Higher (eg. university education), Other, Unknown  If ‘Other’: Specify other education |  |  |
|  | Intellectual disability | | Yes, No, Unknown | Deficit in intellectual and adaptive functioning. Intellectual disability as noted in medical file, or if noted in medical file an IQ equal to or below 70.^46^ |  |
| **A7: Substance use** | | |  |  | Substance use is a risk factor for developing cardiovascular diseases^45^ and may influence the progression of disease. The most common types of used substances are included. |
|  | Tobacco use | | No, Prior, Current, Unknown  > If ‘Prior’: Specify year of smoking cessation | Year someone stopped smoking.  If year is unknown, leave empty. |  |
|  | > If ‘Prior’ or ‘Current’: Tobacco pack years (years) | | Number of pack years | Calculated by multiplying the number of packs of cigarettes smoked per day by the number of years the person has smoked. | PYs are the most common notation of degree of smoking and most often used in research as well. |
|  | eCigarette use | | No, Prior, Current, Unknown  > If ‘Prior’: Specify year of eCigarette use cessation | Year someone stopped using the eCigarette.  If year is unknown, leave empty. |  |
|  | Drug use | | No, Prior, Current, Unknown  > If ‘Prior’: Specify year of drug use cessation | In what year did patient quit drug use.  If year is unknown, leave empty. |  |
|  | > If ‘Prior’ or ‘Current’: Specify quantity/week | | Quantity/unit | Please write the units after the quantity. |  |
|  | > If ‘Prior’ or ‘Current’: Specify type of drug used | | Narcotics, Cannabis, Benzodiazepines, Barbiturates, Sedatives, Hallucinogens, MDMA-derivatives, Other, Unknown  > If ‘Other’: Specify other type of drug used |  |  |
|  | Alcohol use | | No, Prior, Current, Unknown  > If ‘Prior’: Specify year of alcohol use cessation | Alcohol (ab)use is present if noted as such in medical file.  In what year did patient quit alcohol use.  If year is unknown, leave empty. |  |
|  | > If ‘Prior’ or ‘Current’: Specify alcohol quantity per week | | Quantity/unit | Please write the units after the quantity. |  |
| **A8: General health** | | |  |  | To gain understanding of the general health of the patient and to investigate whether this is a risk factor for progression. |
|  | ASA classification | | Normal healthy patient (1), Mild systemic disease (2), Severe systemic disease (3), Severe systemic disease that is a constant threat to life (4), Moribund patient who is not expected to survive without the operation (5), Declared brain dead patient (6), Unknown |  | ASA classification is a widely used classification to assess the health status of patients. Moreover, this is often used by physicians and therefore easily attainable from medical files and across countries as well. |
|  | Ambulatory status | | Fully Ambulatory, Ambulate with Prosthesis, Ambulate with Assistive Device, Wheelchair, Bedbound, Unknown |  | Ambulation = how the majority of walking is carried out. |
|  | Allergies | | Yes, No, Unknown |  | The number of allergies may be an indicator of overall health status; more allergies may indicate a patient’s immune system is prone to immunological diseases. Or, an allergy to certain medication can have an impact on decision-making in medication which in turn may have an impact on progression of cardiovascular disease. |
|  | > If ‘Yes’: Specify allergens | |  | Specify allergen the patient is allergic for. |  |
|  | > If ‘Yes’: Specify grade | | Grade 1: Symptom(s)/sign(s) from one organ system  Grade 2: Symptom(s)/sign(s) from ≥ 2 organ symptoms listed in grade 1  Grade 3: Lower airway  Grade 4: Anaphylaxis, Lower airway  Grade 5: Anaphylaxis, Lower or upper airway | Full description of the grades are provided in the article of Sánchez-Borges et al.^47^ |  |
| **A9: Family history** | | |  |  | Cardiovascular diseases are known to run in certain families, prompting an evaluation of the extent to which genetic predisposition plays a role in development and progression of AAA. |
|  | Family relative | | Mother, Father, Brother, Sister, Grandmother, Grandfather, Child |  |  |
|  | Family history of cardiovascular disease | | No history of cardiovascular disease, Aneurysm, Ruptured aneurysm, Peripheral arterial disease, Myocardial infarction, Heart failure, Cardiovascular disease, Hypertension, Stroke, Diabetes Mellitus, Thrombosis, Malignancy, Yes history of cardiovascular disease (not further specified), Other |  |  |
| **A10: Vital parameters** | | | |  | These factors are included as they are general physical examinations that indicate a patient’s overall physical health status. |
|  | Height (cm) | |  | Collect the examinations that were performed within a timespan of two weeks before or after the index visit (or follow up visit); choose the measurements closest to the date of the index visit (if there are multiple).  If there was no measurement, leave empty. |  |
|  | Weight (kg) | |  | “” |  |
|  | BMI reported in medical file | |  | “” |  |
|  | Blood pressure measured? | | Yes, No, Unknown | “” |  |
|  | > If ‘Yes’: Date of BP measurement | |  | “” |  |
|  | > If ‘Yes’: Measurement position | | Lying down, Sitting, Standing, Unknown |  |  |
|  | > If ‘Yes’: Systolic blood pressure (mmHg) | |  | “” |  |
|  | > If ‘Yes’: Diastolic blood pressure (mmHg) | |  | “” |  |
|  | Heart Rate (bpm) | | > If measured: Specify date of heart rate measurement | “” |  |
|  | Temperature (Celsius) | | > If measured: Specify date of heart rate measurement | “” |  |
| **A11: Imaging data** | | |  |  |  |
|  | Specify IMAGING DATA parameters | |  |  |  |
| **A12: Diagnostic tests** | | |  |  |  |
|  | Specify DIAGNOSTIC TESTS parameters | |  |  |  |
| **A13: Blood test results** | | |  |  |  |
|  | Specify BLOOD TEST RESULTS parameters | |  |  |  |
| **A14: Microbiology test results** | | | |  |  |
|  | Specify MICROBIOLOGY TEST RESULTS parameters | |  |  |  |
| **A15: Medication** | | |  |  |  |
|  | Specify MEDICATION parameters | |  |  |  |
| **A16: Included in other AAA trials/registries/biobank** | | | |  |  |
|  | Participation in other AAA trial/registry/biobank? | | Parel biobank (AUMC), Live biobank (AUMC), Helsinki registry (HUS), Serbia biobank (UOB), Other, Unknown  > If ‘Other’: Specify participation in other AAA trial/registry/biobank |  |  |
| **Other remarks** | | |  |  | **Open field to add remarks/comments/other relevant data** |

| **(2) Relevant data from follow up visit(s)** | | | | | | Relevant information for each defined ‘follow-up visit’. | | |
| --- | --- | --- | --- | --- | --- | --- | --- | --- |
| *Category* | | *Parameter* | *To report* | *Help text* | | *Rationale* | | |
| **MEDICAL REPORT FOLLOW-UP DATA** | | | | | | | | |
| **Change in A1: Did the patient die?** | | | | | |  | |  |
|  | Deceased? | | Yes, No  -obligated question: in case unknown; check death registry- | |  | | If a patient is deceased, follow-up is terminated though follow-up data on cause of death should be filled in. It is a core outcome for AAA | |
|  | > If yes: Date of death | | dd-mm-yyyy | | If date and month are unknown, please enter only the year. | |  | |
|  | > If yes: Cause of death | | AAA-related, Lower limb PAD-related, Other PAD-related, Stroke (ischemic or hemorrhagic), Cardiac related, Other vascular related, Malignancy, Pneumonia, Sepsis, Other, Unknown  > If ‘Other’: Specify other cause of death | | Check death registry if unknown | | Differentiation between vascular- or cardiac-related causes of death. The most common causes are included as answer options. | |
| **Change in A2 and A3: New vascular information?** | | | | | |  | | |
|  | New follow up visit with vascular surgeon, vascular internist, vascular nurse, surgery resident or other medical doctor related to AAA? | | Yes, No  > If ‘Yes’: Specify date of reported follow-up visit related to AAA | If year is unknown, leave empty. | | Follow up visit as defined. | | |
|  | > If ‘Yes’: Specify specialty of physician | | Vascular surgeon or similarly qualified person from the vascular surgery department (such as a vascular nurse, vascular surgery resident, etc), Vascular internist or similarly qualified person from the vascular interal medicine department (such as registered nurse, internal medicine resident, etc.),  General practitioner (GP), (Interventional) Radiologist, Emergency Room (ER) specialist, Other, Unknown  > If ‘Other’: Specify other specialty |  | |  | | |
|  | > If ‘Yes’: specify location of visit | | Emergency department, Outpatient clinic, Inpatient (clinic), Unknown |  | |  | | |
|  | Reason for re-visit with vascular specialist | | Acute admission related to AAA, Pre-operative surveillance, First post-operative control, Post-operative surveillance, Planned admission related to AAA intervention, Referral from another hospital, Visit to the ED (but no admission), Other, Unknown  > If ‘Other’: Specify other reason for re-visit  > If AAA intervention: Specify AAA INTERVENTION parameters | - Acute admission: admission related to AAA was unforeseen. A patient could have been admitted from the emergency department, the outpatient clinic, or from home.  - Surveillance (pre-op): any kind of routine visit to evaluate symptoms, disease progression or to carry out routine measurements (e.g. lab tests or imaging) before any intervention or operation has been performed.  - First post-operative visit: the first postoperative follow-up visits within 3 months after each intervention or operation.  - Post-operative surveillance: visits after the 3 month postoperative period has surpassed and routine visits are performed.  - Planned admission related to AAA intervention: this includes all interventions performed by a vascular surgeon/resident and all interventions performed at request of the vascular surgeon by an interventional radiologist or surgeon of another specialty that is performed in the hospital. This also includes a planned visit of under 24h in which a planned AAA intervention will be performed.  - Referral from another hospital: when the patient is referred from another specialist in another hospital for their disease.  - Visit to the Emergency Departement (ED), but no hospital admission. | | To distinguish between patients that have already undergone an intervention and patients that are under surveillance, to be able to adequately predict disease progression. Aside from routine surveillance, we are also interested in whether patients have been admitted to the hospital in the meantime, whether an intervention has taken place, or whether new pathology has been discovered, to be able to analyze which factors predispose to these. | | |
|  | > If ‘Acute admission’ related to AAA: Specify reason for acute admission | | Symptomatic AAA, (contained) Ruptured AAA, Complications related to previous AAA intervention, Treatment for AAA-related intervention, Other, Unknown  > If ‘Other’: Specify other reason for acute admission  > If ‘Symptomatic AAA’ or ‘(contained) Ruptured AAA’: specify AAA RUPTURE parameters | -Symptomatic AAAs are those presenting with abdominal and/or back pain, tender AAA at palpation, or embolic events, but without breach of the aortic wall.^9^ A symptomatic AAA is an indication of progression of disease and have a higher rupture risk than asymptomatic aneurysms. Progression to a symptomatic aneurysm and/or rupture may be investigated in these patients.  -rAAA is defined as an acute haemorrhage from the AAA outside the true aortic wall with the presence of retroperitoneal and/or intraperitoneal blood.^9^ Rupture indicates aneurysm progression and is a core outcome of AAA. | |  | | |
|  | > If inpatient admission (acute or planned): Complications during in-hospital period? | | Yes, No, Unknown  > If ‘Yes’: Specify date of discharge after admission | If date is unknown, leave empty. | | . | | |
|  | >> If ‘Yes’: Specify complication(s) during in-hospital period and Clavien-Dindo classification | | Fill in for each complication:   - Complications during in-hospital period: Congestive heart failure (decompensatio cordis), Acute heart failure, Cardiac arrhythmia, Myocardial infarction, Atrial fibrillation (AF), Urinary tract infection (UTI), Renal function deterioration, Fever without known cause, Pneumonia, COPD exacerbation, Stroke, TIA, Ileus, Complications related to (endo)graft/bypass, Other, Unknown   > If ‘Other’: Specify other complication   - Clavien-Dindo classification: Grade I, Grade II, Grade IIIa, Grade IIIb, Grade IV, Grade V, Unknown   > Specify other comments about complications (optional). If it is about a specific complication, specify the complication. |  | | [The Clavien-Dindo system](http://www.ncbi.nlm.nih.gov/pmc/articles/PMC1360123/?report=reader) is widely used throughout surgery for grading adverse events (i.e. complications) which occur as a result of surgical procedures.^27^ | | |
|  | Maximum AAA diameter | | Diameter in millimeters | Reported at time of index visit with vascular surgeon. If measured on two modalities for the index visit; report the measurement on CT(A), second best: US, or after, any other. The most recent maximum diameter should be reported.  If someone already has had an AAA intervention, this question does not have to be answered.  If unknown leave it empty. | |  | | |
|  | > Imaging modality of the maximum AAA diameter measurement | | US, CT, CTA (CT Angiography), MRI, MRA (MR Angiography), PET-CT, Other, Unknown  > If 'Other’: Specify other imaging modality | If measured on two modalities for the index visit; report the measurement on CT(A), second best: US, or after, any other. | |  | | |
|  | > Measurement method maximum diameter | | Antero-posterior (AP), CLL (central lumen line) or MPR (multiplanar reconstruction), Other, Unknown  > If 'Other’: Specify other measurement method |  | |  | | |
|  | Did the patient have an AAA intervention before this FU visit? | | Yes, No, Unknown | Previous intervention before index visit with the vascular surgeon. | |  | | |
|  | > If ‘No’: Specify reason no previous AAA intervention | | Below threshold for elective AAA repair, Patient declined surgery, Risk of intervention is too high for patient, Other, Unknown  > If ‘Other’: Specify other reason for no previous AAA intervention | -Below threshold for elective AAA repair (No indication): The treating physician decided that there was no indication for operative treatment, e.g. because disease progression was not severe enough to warrant operative treatment.  -Patient declined intervention: patient chose to not be operated.  -Risk of intervention too high for patient: the risk of intervention was too high because of comorbidities, age, or overall health status of patient. This may be decided by physician or be a decision made through shared-decision making. | |  | | |
|  | > If ‘Yes’: Primary patency after previous intervention? | | Yes, No, Unknown | Definition of patency: “Uninterrupted patency with no occlusion or procedure performed to maintain patency on the stent or native target vessel. Interventions intended to treat endoleak or stent disconnection do not count as loss of primary patency”.^18^ | |  | | |
|  | > If ‘Yes’: Are there any new AAA intervention-related complications since the previous visit? | | No, Yes general AAA intervention-related complications, Yes endograft-related complications, Unknown |  | | Complications are important to note to be able to identify possible risk factors for complications occurring, to be able to prevent them in the future. Moreover, complications may inhibit postoperative recovery and functional status of the patient. | | |
|  | >> If ‘Yes general AAA intervention-related complications’: Specify aneurysm intervention-related complication(s) and Clavien-Dindo classification | | Fill in for each complication:   - Complications intervention-related: Bleeding, Thrombosis, Peripheral embolization, Access site hematoma, Access site seroma (lymphocele), Access site wound infection superficial, Access site wound infection deep, Arterial perforation or rupture, Artery dissection, Renal impairment, Dialysis, Stroke, TIA, Ileus, Gastroparesis, Bowel ischemia, Spinal cord ischemia, Erectile dysfunction, Other, Unknown - Clavien-Dindo classification: Grade I, Grade II, Grade IIIa, Grade IIIb, Grade IV, Grade V, Unknown   > Specify other comments about complications (optional). If it is about a specific complication, specify the complication. |  | |  | | |
|  | >> If ‘Yes endograft-related complications’: Specify endograft-related complication(s) and Clavien-Dindo classification | | Fill in for each complication:   - Complications endograft-related: (Endo)graft infection, (Endo)graft occlusion, (Endo)graft migration, Device erosion through aortic or iliac wall, Buttock/leg claudication/ischemia, Endoleak, Other, Unknown - Clavien-Dindo classification: Grade I, Grade II, Grade IIIa, Grade IIIb, Grade IV, Grade V, Unknown   > Specify other comments about complications (optional). If it is about a specific complication, specify the complication. |  | |  | | |
|  | >>> If ‘Endoleak’: Specify type of Endoleak | | No, Type I, Type Ia, Type Ib, Type II, Type III, Type IV, Type V, Unknown | If not applicable, leave empty. | |  | | |
|  | >>> If ‘Endoleak’: Specify if Endoleak is persistent or new | | Persistent, New, Unknown | If not applicable, leave empty. | |  | | |
|  | Any AAA-related changes (for better or for worse)? | | Yes, No  > If ‘Yes’: Specify changes *[so update only the new/changed parameters in this section (A2) that are reported in a new report]* | If there are changes (for better or for worse, i.e. also if the symptoms improved after a surgery or interventions) in the AAA-related information, we ask you to fill in the parameters that have changed since the last visit. | |  | | |
|  | Any general vascular-related changes (for better or for worse)? | | Yes, No  > If ‘Yes’: Specify changes *[so update only the new/changed parameters in this section (A3) that are reported in a new report]* | If there are changes (for better or for worse, i.e. also if the symptoms improved after a surgery or interventions) in the vascular-related information, we ask you to fill in the parameters that have changed since the last visit. | |  | | |
| **Change in A4: New cardiac report after the previous visit with vascular surgeon?** | | | | | |  | | |
|  | New follow up visit with cardiologist, cardiac nurse, cardiac surgeon, cardiologist, or any other cardiac specialist (such as qualified resident, registered nurse or technician), since the last follow up visit? | | Yes, No  > If ‘Yes’: Specify date last-reported cardiac visit |  | |  | | |
|  | > If ‘Yes’: Specify location of index visit | | Emergency department, Outpatient clinic, Inpatient (clinic), Unknown |  | |  | | |
|  | > If ‘Yes’: Specify reason for (re)visit with cardiac specialist | | Newly diagnosed, Acute cardiac-related admission, Pre-operative surveillance, First post-operative control, Post-operative surveillance, Planned admission related to cardiac intervention, Referral from another hospital, Visit to the ED (but no admission), At patient's request because of new or worsening symptoms, Preoperative screening for AAA surgery/intervention, Other, Unknown  > If ‘Planned admission’: Specify OTHER SURGICAL INTERVENTION parameters  > If ‘Other’: Specify other reason for (re)visit with cardiac specialist | -Newly-diagnosed: specifically first ever visit to the vascular surgeon with a new diagnosis of a new cardiac disease.  -Acute cardiac-related admission: admission was unforeseen. A patient could have been admitted from the emergency department, the outpatient clinic, or from home.  -Surveillance (pre-op): any kind of routine visit to evaluate symptoms, disease progression or to carry out routine measurements (e.g. lab tests or imaging) before any intervention or operation has been performed.  -Post-operative: the first postoperative follow-up visits within 3 months after each intervention or operation.  -Post-operative surveillance: visits after the 3 month postoperative period has surpassed and routine visits are performed.  -Planned admission related to cardiac intervention: this includes all interventions performed by a cardiologist, cardiac surgeon, or any intervention requested by cardiologist that is performed in the hospital. This also includes a planned visit of under 24h in which a planned cardiac intervention will be performed. Planned admission: patient and treating physician were aware of admission occurring at least 24 hours in advance. Examples of planned admission are: pre-operative or pre-intervention.  -Referral from another hospital: when the patient is referred from another specialist in another hospital for their disease.  - Visit to the Emergency Department (ED), but no hospital admission.  -At patient’s request because of new or worsening symptoms: visits that are scheduled additionally or earlier than routine visits because the patient experiences disease progression.  -Preoperative screening for a AAA surgery/intervention: visit for a cardiac screening before a AAA-related surgery or intervention. | |  | | |
|  | > If ‘Acute cardiac admission’: Specify reason | | Myocardial infarction, Cardiac arrhythmia, Congestive heart failure, Other, Unknown  > If ‘Other’: Specify other reason acute cardiac admission  > Specify date of discharge after planned admission | If unknown, leave date empty. | |  | | |
|  | > If inpatient admission (acute or planned): Complications during in-hospital period? | | Yes, No, Unknown |  | |  | | |
|  | >> If ‘Yes’: Specify complications in-hospital period and Clavien-Dindo classification | | Fill in for each complication:   - Complications during in-hospital period: Congestive heart failure (decompensatio cordis), Acute heart failure, Cardiac arrhythmia, Myocardial infarction, Atrial fibrillation (AF), Urinary tract infection (UTI), Renal function deterioration, Fever without known cause, Pneumonia, COPD exacerbation, Stroke, TIA, Ileus, Complications related to (endo)graft/bypass, Other, Unknown   > If ‘Other’: Specify other complication   - Clavien-Dindo classification: Grade I, Grade II, Grade IIIa, Grade IIIb, Grade IVa, Grade IVb, Grade V   > Specify other comments about complications in general (optional). If it is about a specific complication, specify the complication. | [The Clavien-Dindo system](http://www.ncbi.nlm.nih.gov/pmc/articles/PMC1360123/?report=reader) is widely used throughout surgery for grading adverse events (i.e. complications) which occur as a result of surgical procedures.^27^ | |  | | |
|  | Reported changes in A4: Cardiac history? | | Yes, No, Unknown  > If ‘Yes’: Specify what and date *[so update only the new/changed parameters in this section (A4) that are reported in a new report]* |  | |  | | |
| **Change in A5: Comorbidities?** | | | |  | |  | | |
|  | If change in Comorbidities: specify changes | | *Update only the reported new/changed parameters in this section (A5)* |  | |  | | |
| **Change in A6: Social and economic status?** | | | |  | |  | | |
|  | If change in Social and economic status: specify changes | | *Update only the reported new/changed parameters in this section (A6)* |  | |  | | |
| **Change in A7: Substance use?** | | | |  | |  | | |
|  | If change in Substance use: specify changes | | *Update only the reported new/changed parameters in this section (A7)* |  | |  | | |
| **Change in A8: General Health?** | | | |  | |  | | |
|  | If change in General Health: specify changes | | *Update only the reported new/changed parameters in this section (A8)* |  | |  | | |
| **Change in A9: Family history?** | | | |  | |  | | |
|  | If change in Family history: specify changes | | *Update only the reported new/changed parameters in this section (A9)* |  | |  | | |
| **Change in A10: Vital parameters?** | | | | | |  | | |
|  | If change in Vital parameters: specify changes | | *Update only the reported new/changed parameters in this section (A10)* |  | |  | | |
| **Change in A11: Imaging data?** | | | | | |  | | |
|  | If change in Imaging data: specify changes | | *Update only the reported new/changed parameters in this section (A11)* |  | |  | | |
| **Change in A12: Diagnostic tests?** | | | | | |  | | |
|  | If change in Diagnostic tests: specify changes | | *Update only the reported new/changed parameters in this section (A12)* |  | |  | | |
| **Change in A13: Blood test results?** | | | | | |  | | |
|  | If change in Blood test results: specify changes | | *Update only the reported new/changed parameters in this section (A13)* |  | |  | | |
| **Change in A14: Microbiology test results?** | | | | | |  | | |
|  | If change in Microbiology test results: specify changes | | *Update only the reported new/changed parameters in this section (A14)* |  | |  | | |
| **Change in A15: Medication?** | | | | | |  | | |
|  | If change in Medication: specify changes | | *Update only the reported new/changed parameters in this section (A15)* |  | |  | | |
| **Change in A16: New inclusion in other AAA trials/registry/biobank?** | | | | | |  | | |
|  | If change in New inclusion in other AAA trials/registry/biobank: specify changes | | *Update only the changed parameters in this section (A16) that are reported* |  | |  | | |
| **Other remarks FU** | | |  |  | | Open field to add remarks/comments | | |

| **(3) Events** | | | | |  | All relevant information regarding AAA rupture, AAA interventions, other surgical interventions. |
| --- | --- | --- | --- | --- | --- | --- |
| *Category* | | *Parameter* | *To report* | *Help text* | | *Rationale* |
| **AAA RUPTURE parameters** | | |  | |  |  |
|  | Date and time of presentation at the Emergency Department (ED) (at the index hospital) | |  | Time of arrival at the hospital that is participating in this study (A patient may arrive at another hospital first and is referred to another).  If date is unknown, leave empty. | | Time of arrival at the hospital that is participating in this study (A patient may arrive at another hospital first and is referred to another). |
|  | Was the patient first brought to a different hospital? | | Yes, No, Unknown |  | | In some cases patients will be admitted to another hospital first. When it becomes clear the patient has a rupturing aneurysm, the patient can be transferred to another hospital for treatment. This influences the time of arrival to intervention, and, in rupturing aneurysms time is key so this is a very important parameter for disease outcome. |
|  | Was it a (contained) rupture at time of arrival in this hospital? | | Yes, No, Unknown | -rAAA is defined as an acute haemorrhage from the AAA outside the true aortic wall with the presence of retroperitoneal and/or intraperitoneal blood.  -A contained rAAA is when the haematoma is temporarily sealed by the retro-peritoneum. | | A contained rAAA is when the haematoma is temporarily sealed by the retro-peritoneum.^9^ A contained rupture often leads to less severe bleeding, but can proceed to become a regular aneurysm rupture. |
|  | GCS-EMV score at presentation ED | | Specify EMV score: E(1-4), M(1-5), V(1-6) |  | | Indication of patient’s consciousness when presented at the ED. |
|  | Systolic blood pressure (RR) at presentation ED (mmHg) | |  | It refers to the first measurement of RR in the ED. | | Indicates whether the patient is hemodynamically stable or unstable at presentation with a ruptured AAA at the ER. It refers to the first measurement of RR. |
|  | Diastolic blood pressure (RR) at presentation ED (mmHg) | |  | It refers to the first measurement of RR in the ED. | | Indicates whether the patient is hemodynamically stable or unstable at presentation with a ruptured AAA at the ER. It refers to the first measurement of RR. |
|  | Heart rate (HR) at presentation ED (bpm) | |  | It refers to the first measurement of HR in the ED. | | Indicates whether the patient is hemodynamically stable or unstable at presentation with a ruptured AAA at the ER. It refers to the first measurement of HR. |
|  | Died in the ED? | | Yes, No  > If ‘Yes’: Specify Mortality-related information at *‘Change in A1: Did the patient die?’* |  | |  |
|  | Was acute surgery/intervention performed on the ruptured/symptomatic aneurysm? | | Yes, No, Unknown  > If ‘Yes’: Specify AAA INTERVENTION parameters |  | |  |
|  | If ‘No’: Specify reason for no acute intervention | | Unfit for surgery, Wishes of patient (and/or relatives), No rupture (symptomatic AAA), Other, Unknown |  | | - Unfit for surgery: as determined by the treating physicians, e.g. because of comorbidities or too hemodynamically unstable at presentation in the ED. - The patient (and/or relatives) do not wish to have surgery anymore (for example because of poor prognostics).   To evaluate what factors are important in decision-making to undergo an intervention for rAAA. |
| **AAA INTERVENTION parameters** | | | | |  | Inventory of each AAA-related intervention performed. For each procedure, report technical procedural information of the intervention.  These parameters can be subdivided into:   - Type of intervention and technicalities related to the intervention that is undergone. There are multiple treatment strategies, multiple brands making technical equipment for vascular procedures, and within the technical equipment multiple possibilities concerning size, diameter, length, etc. To be able to identify which intervention is carried out exactly, all these specifics should be known. Also, type of intervention and its technical specifics may influence the postoperative course of AAA. - OR-specific information: general OR-related information such as blood loss, complications, and ml of contrast used. These all influence postoperative course. |
| **Procedure** | | |  | |  |  |
|  | Date of the current AAA-related intervention in this hospital? | | dd-mm-yyyy | Of intervention/procedure in this hospital.   If date is unknown, leave empty.  If multiple procedures in a few days, please fill in characteristics of these procedures separately (in a new 'AAA intervention' field). | |  |
|  | Reason for AAA-related intervention | | AAA diameter over threshold for intervention (according to ESVS guidelines), Symptomatic AAA, Ruptured AAA, Contained AAA rupture, Penetrating Aorta Ulcus (PAU), Inflammatory aneurysm, Reintervention because infection of in-situ prosthesis, Reintervention because of persisting endoleak, Reintervention because of disease progression, Other, Unknown  > If ‘Other’: Specify other reason for AAA-related intervention | - The threshold for considering elective abdominal aortic aneurysm repair is recommended to be >5.5 cm diameter for men and >5.0 diameter for women. Or, when the aneurysm grows >1cm/year.^9^  - Symptomatic AAAs are those presenting with abdominal and/or back pain, tender AAA at palpation, or embolic events, but without breach of the aortic wall.^9^  - rAAA is defined as an acute haemorrhage from the AAA outside the true aortic wall with the presence of retroperitoneal and/or intraperitoneal blood.^9^ - A contained rAAA is when the haematoma is temporarily sealed by the retro-peritoneum.^9^ - PAU: Defined as ulceration of an atherosclerotic plaque that penetrates through the aortic intima resulting in a variable amount of haematoma within the aortic wall - An implanted prosthesis may get infected and may need to be removed. - Persisting endoleak: An endoleak causing the aneurysm to progress may require additional intervention. -Reintervention because of disease progression: as determined by physician. Example: continuing dilatation of the aneurysm wall. | | To identify reason of intervention for AAA. |
|  | (contained) AAA rupture at time of procedure? | | No, Yes ruptured AAA, Yes contained AAA rupture, Unknown | -rAAA is defined as an acute haemorrhage from the AAA outside the true aortic wall with the presence of retroperitoneal and/or intraperitoneal blood.  -A contained rAAA is when the haematoma is temporarily sealed by the retro-peritoneum.^9^ | |  |
|  | Procedure type | | Open surgical repair Endovascular repair, Planned two-stage procedure, Revision after previous AAA repair, Other, Unknown  > If ‘Other’: Specify other procedure type | Multiple options can be filled in if two types are used within one intervention.  If surgery was converted from endovascular procedure to open, you should select both the endovascular procedure and open surgery.  If the intervention was part of a two-stage procedure, select the endovascular procedure that you’re currently filling in and select ‘two-stage procedure’.  If the surgery was a revision of a previous AAA surgery, select this option. | | Type of intervention is noted to be able to evaluate which treatment was performed.  Multiple options can be filled in if two types are used within one intervention.  If surgery was converted from endovascular procedure to open, you should select both the endovascular procedure (for example, EVAR) and select ‘conversion to open surgery’.  If the intervention was part of a two-stage procedure, select the endovascular procedure that you’re currently filling in *and* select ‘two-stage procedure’. |
|  | > If ‘Endovascular repair’: Specify endovascular procedure type | | EVAR, BEVAR, FEVAR, chEVAR, Other, Unknown  > If ‘Other’: Specify other endovascular procedure type | Multiple options can be filled in if two types are used within one intervention. | |  |
|  | >> Type of main device | | Medtronic Talent, Medtronic Endurant, Medtronic Endurant II/IIs, Gore Excluder, Gore IBE, Endologix AFX, Endologix Nellix, Endologix Ovation iX, Endologix Alto, Cook Zenith flex, Cook Zenith Alpha, Cook Zenith Fenestrated, Cook Zenith Branch Endovascular Graft - Iliac Bifurcation (ZBIS), Cook custom made fenestrated, Cook T-branch off-the-shelf, Terumo Aortic Treo, Terumo Aortic Anaconda, Artivion(/Jotec) E-nside off-the-shelf inner branch, Artivion(/Jotec) E-xtra Design custom inner branch, Artivion(/Jotec) E-xtra Design custom outer branch, Other, Unknown  > If ‘Other’: Specify other type of main device |  | | We named the most brands of main devices to be able to identify between different types of stent types and materials. These may or may not be factors predisposing to treatment success. |
|  | >> Brand of main device | | Medtronic, Gore, Endologix, Cook Medical, Terumo, Artivion/Jotec, Other, Unknown  > If ‘Other’: Specify other brand of main device |  | |  |
|  | >> Size of main device (diameter) | |  | In mm. | |  |
|  | >> How many fenestrations(fen)/scallop(s)? | | None, 4 fen, 3 fen, 2 fen, 1 fen, 3 fen + 1 scallop, 2 fen + 1 scallop, 1 fen + 1 scallop, 1 scallop, Other, Unknown  > If ‘Other’: Specify how many fenestrations/scallop(s) |  | |  |
|  | >> Number of bridging stent(s) used | | 0, 1, 2, 3, 4, 5, Unknown |  | |  |
|  | >>> If not ‘0’ or ‘Unknown’: Specify type of bridging stent | | Advanta V12/iCast (Atrium medical/Getinge), Viabahn VBX Gore, Viabahn SE Gore, Begraft (Bentley), E-ventus (Jotec), LifeStream (BD), Covera (BD), Wallgraft (Boston Scientific), Other, Unknown  > If ‘Other’: Specify other type of bridging stent |  | |  |
|  | >>> If not ‘0’ or ‘Unknown’: Specify brand of bridging stent | | Atrium medical/Getinge, Gore, Bentley Innomed, Artivion/Jotec, Becton Dickinson and Company (BD)/CR Bard, Boston Scientific, Other, Unknown  > If ‘Other’: Specify other brand of bridging stent |  | |  |
|  | >>> If not ‘0’ or ‘Unknown’: Specify details of bridging stents (diameter, length, and artery) | | Diameter,  Length,  Artery; Celiac trunk, Superior mesenteric artery (SMA), Left renal artery (LRA), Right renal artery (RRA), Accessory renal artery, Unknown |  | |  |
|  | >> Endoanchors used? | | Yes, No, Unknown |  | |  |
|  | >> Cuff used? | | Yes, No, Unknown |  | |  |
|  | >>> If ‘Yes’: Specify brand of cuff | | Medtronic, Gore, Terumo, Artivion/Jotec, Cook Medical, Endologix, Other, Unknown  > If ‘Other’: Specify other brand of cuff |  | |  |
|  | >>> If ‘Yes’: Specify size of cuff (diameter and length) | | Diameter,  Length |  | |  |
|  | >> Other graft/implant used? | | Yes, No, Unknown  > If ‘Yes’: Specify other graft/implant used |  | |  |
|  | > If ‘Open’ procedure: Specify open procedure type. | | Open surgical repair (primary), Open surgical revision (after previous AAA intervention), Conversion to open surgery, Other, Unknown  > If ‘Other’: Specify other open procedure type | Multiple options can be filled in if two types are used within one intervention. | |  |
|  | >> Type of graft material | | Textile polyester (Dacron), ePTFE, Other, Unknown  > If ‘Other’: Specify other type of graft material |  | |  |
|  | >> Antimicrobial substance used? | | No, Yes silver, Yes rifampicin coating, Yes other, Unknown  > If ‘Yes other’: Specify other antimicrobial substance |  | |  |
|  | > If applicable: type of main device (if known) | | Medtronic Talent, Medtronic Endurant, Medtronic Endurant II/IIs, Gore Excluder, Gore IBE, Endologix AFX, Endologix Nellix, Endologix Ovation iX, Endologix Alto, Cook Zenith flex, Cook Zenith Alpha, Cook Zenith Fenestrated, Cook Zenith. Branch Endovascular Graft - Iliac Bifurcation (ZBIS), Cook custom made fenestrated, Cook T-branch off-the-shelf, Terumo Aortic Treo, Terumo Aortic Anaconda, Artivion(/Jotec) E-nside off-the-shelf inner branch, Artivion(/Jotec) E-xtra Design custom inner branch, Artivion(/Jotec) E-xtra Design custom outer branch, Other, Unknown  > If ‘Other’: Specify other type of main device |  | | Same as above, and the type can be indicative for the extent of disease ( e.g. involvement of visceral arteries or not) |
|  | Technical success | | Yes, No, Unknown | For EVAR: primary technical success is defined on an intent-to-treat basis and requires the successful introduction and deployment of the device in the absence of surgical conversion or mortality, type I or III endoleaks*, or graft limb obstruction. '*' visible on final angiography right after procedure in the OR  For OSR: Primary technical success for open surgical repair should be reported on an intent-to-treat basis and should require replacement or bypass of the aneurysmal segment with a prosthetic graft in the absence of mortality or graft thrombosis either during surgery or during the initial 24-hour postoperative period.^19^ | |  |
|  | Residual endoleak? | | No, Type I, Type Ia, Type Ib, Type II, Type III, Type IV, Type V, Unknown | Residual endoleak visible on final angiography right after procedure in the OR.  Multiple options possible. | |  |
|  | (Non-fatal) complications during intervention? | | Yes, No, Unknown |  | |  |
|  | > If ‘Yes’: Specify complications during surgery/intervention (non-fatal) and Clavien-Dindo classification (see classification below) (Only fill in the amount of complications that were there, the other fields can be left empty) | | Fill in for each complication:   - Complications during surgery/intervention: Arterial bleeding, Venous bleeding, Peripheral embolization, Thrombosis, Cardiac related (eg. cardiac arrest), Hemorrhagic shock, Distributive shock, Obstructive shock, Stroke, TIA, Complications related to (endo)graft/bypass, Other, Unknown   > If ‘Other’: Specify other complication   - Specify Clavien-Dindo classification: Grade I, Grade II, Grade IIIa, Grade IIIb, Grade IV, Grade V, Unknown   > If ‘Other’: Specify other comments about complications (optional). If it is about a specific complication, specify the complication. | Major arterial or venous bleeding: as noted by surgeon in the operation report. This includes only major bleeding that were controlled with difficulty, not bleeding from vessels that could be easily managed with stitches or coagulation. | | Complications during operation/intervention influence postoperative recovery. |
|  | Death during surgery? | | Yes, No  > If ‘Yes’: Specify Mortality-related information at *‘Change in A1: Did the patient die?’* |  | | The exact cause of death must be defined at the cause of death section. |
|  | Blood loss (mL) | |  |  | | Heavy blood loss during operation has an impact on postoperative recovery. |
|  | Total procedure time: start-end incision (min) | |  | Total procedure time is defined as the moment from start of incision by the surgeon until closing of the skin. Hence, start surgical time until stop surgical time. | |  |
|  | Amount of contrast used (ml) | |  |  | | Contrast is harmful especially for the kidneys and impaired kidney function hinders postoperative recovery. Therefore, to evaluate postoperative kidney function, contrast given during the procedure is an important measure. |
|  | X-ray exposure time (min) | |  |  | | This could be a measure of how complicated the procedure is (long exposure time means that for example the anatomy is challenging). |
| **After intervention (in-hospital)** | | | | |  | To identify the severity of the complications (if any), or the postoperative recovery period. These parameters can be noted during the in-hospital period. If a complication occurs after this period, a new follow-up visit should be filled in. |
|  | Days at PACU (Post Anesthesia Care Unit) after the intervention | |  | Calculate based on the date. | |  |
|  | Days at ICU (intensive care unit) after the intervention | |  | Calculate based on the date. | | Days in ICU treatment indicate how much support a patient needs and therefore is an indication of postoperative systemic condition |
|  | Days at MCU (medium care unit) after the intervention | |  | Calculate based on the date. | | Same as above, but then in terms of medium care |
|  | Days at vascular surgery ward after the intervention | |  | Calculate based on the date. | | A longer stay in the surgery ward is also an indication of postoperative systemic condition, moreover it predisposes to additional comorbidities such as infection (pneumonia) |
|  | In-hospital mortality (after procedure?) | | Yes, No  > If ‘Yes’: Specify Mortality-related information at *‘Change in A1: Did the patient die?’* |  | |  |
|  | Date of discharge from hospital | |  | If date is unknown, leave empty. | | To determine the total postoperative hospital stay |
|  | Duration of total admission (days) | |  | Calculate based on the date. | |  |
|  | Insert the first measured vital parameters after the procedure | | Specify under VITAL PARAMETERS | If unknown, leave empty. | |  |
|  | Discharge destination | | Home independently, Home with help, Nursery home, Living with relatives, Other hospital, Other, Unknown  > If ‘Other’: Specify other discharge destination |  | | Living at home without the need of additional help indicates that a person is at least self-sufficient in their daily needs. Indicative for frailty after intervention.  Home with help may be help from government-assisted facilities such as a nurse or help from relatives. |
|  | Are there any new AAA intervention-related complications since the previous visit? | | No, Yes general AAA intervention-related complications, Yes endograft-related complications, Unknown |  | | Complications are important to note to be able to identify possible risk factors for complications occurring, to be able to prevent them in the future. Moreover, complications may inhibit postoperative recovery and functional status of the patient. |
|  | > If ‘Yes’: Specify AAA intervention-related complications and Clavien-Dindo classification | | Fill in for each complication:   - Complications intervention-related: Bleeding, Thrombosis, Peripheral embolization, Access site hematoma, Access site seroma (lymphocele), Access site wound infection superficial, Access site wound infection deep, Arterial perforation or rupture, Artery dissection, Renal impairment, Dialysis, Stroke, TIA, Ileus, Gastroparesis, Bowel ischemia, Spinal cord ischemia, Erectile dysfunction, Other, Unknown - Clavien-Dindo classification: Grade I, Grade II, Grade IIIa, Grade IIIb, Grade IV, Grade V, Unknown   > Specify other comments about complications (optional). If it is about a specific complication, specify the complication. |  | | [The Clavien-Dindo system](http://www.ncbi.nlm.nih.gov/pmc/articles/PMC1360123/?report=reader) is widely used throughout surgery for grading adverse events (i.e. complications) which occur as a result of surgical procedures.^27^ |
|  | >> If ‘Yes: endograft-related complications’, specify endograft-related complication(s) and Clavien-Dindo classification | | (Fill in for each complication:   - Complications endograft-related: (Endo)graft infection, (Endo)graft occlusion, (Endo)graft migration, Device erosion through aortic or iliac wall, Buttock/leg claudication/ischemia, Endoleak, Other, Unknown   > If ‘Other’: Specify other complication   - Clavien-Dindo classification: Grade I, Grade II, Grade IIIa, Grade IIIb, Grade IVa, Grade IVb, Grade V   > Specify other comments about complications (optional). If it is about a specific complication, specify the complication. |  | |  |
| **OTHER SURGICAL INTERVENTIONS** | | | | |  |  |
|  | Specify if it was a (non-cardiac) vascular surgery/intervention (in another hospital) | | Yes, No |  | |  |
|  | > If ‘Yes’: Specify previous surgery/intervention that is performed | | Amputation, Angiography performed but treatment not successful because of occlusive stenosis (peripheral), Atherectomy (peripheral), Balloon angioplasty (percutaneous transluminal angioplasty) (including drug-coated balloons) (peripheral), Endarterectomy (thromboendarterectomy) (peripheral), Endarterectomy (thromboendarterectomy) (carotid), Open bypass surgery (peripheral), Stenting (peripheral), EVAR, FEVAR, BEVAR, ChEVAR, Open surgical AAA repair, Other, Unknown  > If ‘Other’: Specify other vascular intervention | Only choose multiple options if they were performed within the same intervention. If it were two separate interventions; make a new 'surgical intervention' data entry. | |  |
|  | >> If ‘Evar’: Type of main device | | Medtronic Talent, Medtronic Endurant, Medtronic Endurant II/IIs, Gore Excluder, Gore IBE, Endologix AFX, Endologix Nellix, Endologix Ovation iX, Endologix Alto, Cook Zenith flex, Cook Zenith Alpha, Cook Zenith Fenestrated, Cook Zenith Branch Endovascular Graft - Iliac Bifurcation (ZBIS), Cook custom made fenestrated, Cook T-branch off-the-shelf, Terumo Aortic Treo, Terumo Aortic Anaconda, Artivion(/Jotec) E-nside off-the-shelf inner branch, Artivion(/Jotec) E-xtra Design custom inner branch, Artivion(/Jotec) E-xtra Design custom outer branch, Other, Unknown  > If ‘Other’: Specify other type of main device |  | |  |
|  | >> If ‘Evar’: Brand of main device | | Medtronic, Gore, Endologix, Cook Medical, Terumo, Artivion/Jotec, Other, Unknown  > If ‘Other’: Specify other brand of main device |  | |  |
|  | Specify if it was another (not vascular) surgery/intervention | | Yes, No | Only choose multiple options if they were performed within the same intervention. If it were two separate interventions; make a new 'surgical intervention' data entry. | |  |
|  | > If ‘Yes’: Specify previous surgery/intervention that is performed | | Ablation, AV-Block, Defibrillator (ICD), Pacemaker, CABG, Percutaneous Coronary Intervention (Coronary Angioplasty or Stenting), Cholecystectomy, Appendectomy, Cataract surgery, Other, Unknown  > If ‘Other’: Specify other vascular intervention |  | |  |
|  | Date of previous surgery | |  | If date is unknown, leave empty. | |  |

| **(4) Repeated data** | | | | | | | | The following are repeated measures and are important to track over time to monitor the growth and progression of AAA and cardiovascular events (standard measurements such as blood pressure are indicative for the health status of the patient). |
| --- | --- | --- | --- | --- | --- | --- | --- | --- |
| *Category* | | *Parameter* | | | *To report* | *Help text* | | *Rationale* |
| **IMAGING DATA** | | | | |  | | | All available imaging of the patient related to cardiovascular disease (abdominal, extremities, thoracic, cranial) (also all cardiovascular imaging before the index visit). All data related to the imaging can be included such as dicoms, photos, videos, text or PDF reports. |
|  | Add all available imaging related to cardiovascular disease (abdominal, extremities, thoracic, cranial). Also imaging for another indication than cardiovascular disease, but which contain either abdomen, extremities, thorax, or cranium. | | | | Dicoms, Images, videos and reports (text, pdfs, other) |  | |  |
|  | Date of imaging examination | | | |  |  | |  |
|  | Imaging modality | | | | > If ‘Other’: Specify other Imaging modality |  | |  |
|  | Add written report of imaging (copy text) | | | |  |  | |  |
|  | (or) add written report of imaging (upload file) | | | |  |  | |  |
| **DIAGNOSTIC TESTS** | | | | |  | | | All available diagnostics of the patient related to cardiovascular disease (abdominal, extremities, thoracic, cranial) should be included. All data can be included such as ECG transcripts, ABI/TBI tests, photos, videos, text or PDF reports of other tests. |
| **Ankle Brachial Index (ABI)** | | | | |  | | |  |
|  | Date examination was performed | | | |  |  | |  |
|  | Ankle/Brachial Index (ABI) highest arm blood pressure | | | |  | If unknown, leave empty. | | Ankle-brachial index (ABI): calculated ratio between the systolic BP measured at the ankle (in the anterior tibial or dorsalis pedis artery and the posterior tibial artery, retaining the higher value) and the brachial systolic BP (measured in both arms, retaining the higher value).^42^  ABI is an established diagnostic criteria for diagnosis of peripheral arterial disease and indicates disease severity. Moreover, its use as a diagnostic is recommended by the SFMV/SCVE 2021 guidelines.^42^ |
|  | ABI highest ankle blood pressure: Right | | | |  | If unknown, leave empty. | |  |
|  | ABI highest ankle blood pressure: Left | | | |  | If unknown, leave empty. | |  |
|  | AB Index (if the blood pressures are unknown) | | | |  | If unknown, leave empty. | |  |
|  | TBI: highest toe pressure: Right | | | |  | If unknown leave empty. | | Toe pressure is a measure of the blood pressure in one of the digits, most often the hallux. It is a recommended diagnostic measure in PAD.^42^  This variable will also be used to measure the toe/brachial index (TBI), using also the highest arm blood pressure. TBI is a measure of the blood pressure in one of the digits, most often the hallux, relative to the brachial pressure. It is a recommended diagnostic measure in PAD.^42^ |
|  | TBI: highest toe pressure: Left | | | |  | If unknown leave empty. | |  |
|  | TB Index (if the blood pressures are unknown) | | | |  | If unknown, leave empty. | |  |
|  | Upload available report from the ABI/TBI measurement | | | |  |  | |  |
| **ECG** |  | | | |  | | | ECG informs us about cardiac status. Will provide information on how the ECG might have changed compared to previous visits, preoperative vs. postoperative, etc. Specify date of test performed. |
|  | Date of ECG | | | |  | |  |  |
|  | Pathological ECG patterns (copy report) | | | |  | |  |  |
|  | Upload ECG examination file | | | |  | |  |  |
| **LABORATORY AND MICROBIOLOGY TESTS** | | | | | | | | We want to collect a complete blood count to assess the relation of blood values and disease progression |
| **Blood tests** | | |  | |  |  | |  |
|  | Date of blood sample collection | | | |  |  | |  |
|  | Reason of blood collection | | | |  | Include the reason/circumstances (acute admission, routine check-up, etc) for the lab test, to enable us to correlate the results with the patient’s health status at that particular moment. | |  |
|  | Specialty of physician that requested blood collection | | | |  |  | |  |
|  | Remarks of blood collection | | | |  | Other remarks for the blood test | | For example, reason for the blood test, by which department it was requested, taken at GP, etc. |
|  | Upload blood test results | | | |  |  | |  |
|  | Prefer manual input of each laboratory test result? | | | | Yes, No |  | |  |
|  | > If ‘Yes’: Laboratory blood test results (all tested parameters):  > Hemoglobin concentration (mmol/L)  > Hematocrit value (L/L)  > Blood white blood cell count (*10^9/L)  > Blood trombocyte count (*10^9/L)  > Blood Erythrocyte sedimentation rate (ESR) (mm/hour)  > Blood urea (mmol/L)  > Blood creatinine (umol/L)  > Blood CRP (mg/L)  > Blood glucose (mmol/L)  > Blood glucose fasting (mmol/L)  > Blood HbA1c (mmol/mol)  > Sodium (mmol/L)  > Potassium (mmol/L)  > eGFR (mL/min/1.73m2)  > Blood total cholesterol (mmol/L)  > Blood HDL-cholesterol (mmol/L)  > Blood LDL-cholesterol (mmol/L)  > Blood triglycerides count (mmol/L)  > Blood Hs Troponine T (ng/L)  > Repeated measurment Blood Hs Troponine T (ng/L)  > Blood BNP count (ng/L) | | | |  |  | |  |
| **Microbiology tests** | | | |  |  | |  |  |
|  | Date of microbiology sample collection | | | |  | |  |  |
|  | In what circumstances was the sample taken? | | | | During OR, At the ward, At the outpatient clinic, Other, Unknown | |  |  |
|  | What micro-organism or virus was found in the sample? | | | |  | | If multiple, report multiple. | This variable is included to evaluate whether there are micro-organisms present in the sample, to evaluate whether these are treated properly and whether they might be a risk factor for progression of AAA. Bacteremia is an indicator for severity of the infection and may be prognostic of deterioration. |
|  | In what type of sample was this tested? | | | | Blood culture, Wound, Urine, Bone, Abscess aspiration, Other, Unknown  > If ‘Other’: Specify in what other type of sample this was tested | | If multiple, check multiple. |  |
|  | > If ‘Wound’: what was the location of this wound? | | | | Toes (dig 1-5) R, Toes (dig 1-5) L, Forefoot R, Forefoot L, Ankle R, Ankle L, Lower limb R, Lower limb L, Knee R, Knee L, Above knee R, Above knee L, Groin R, Groin L, Midline laparotomy, Other, Unknown | | If multiple, check multiple. |  |
| **MEDICATION RECORDS** | | | | |  | | | It is crucial to know what cardiovascular disease management medication the patient takes when analyzing AAA disease progression and cardiovascular events. Medication can provide information about the extent of disease or perhaps reveal (over- or) under treatment of patients.  Moreover, quitting a certain medication may predispose to adverse events, e.g. increased blood pressure if a beta blocker is stopped. Therefore, we want to keep track of medication use.  For medications, we subdivided them into the most common types of antihypertensive, lipid-lowering, anticoagulant, anti-arrhythmic, and blood glucose-lowering medication, in addition to immunosuppressive (Corticosteroid, immune suppressive) medication and benzodiazepines (the latter because it’s an indicator of (mental) health) and opiates (because the use of these gives an indication of how much pain is experienced) |
|  | Upload Medication report file | | | |  | |  |  |
|  | Current medication types at the time of the visit | | | | ACE inhibitor  Alpha-Blocker  Amiodarone/Cardarone  Angiotensin receptor blocker  Benzodiazepine  Beta-Blocker  Calcium antagonist  Corticosteroids  Digoxin  Direct oral anticoagulation (DOAC)/NOAC  Diuretics  Immune suppressive  Insulin  Nitrate  Opioids  Oral blood glucose lowering medicine  Other lipid lowering medicine  PCSK9 inhibitor  Statins  Thrombocyte aggregation inhibitor  Vitamin K antagonist/coumarin derivatives  Antibiotics  Other medication  Unknown  > If ‘Other medication’: Specify other medication | | Report the medication types that are prescribed to the patient at the time of the visit. |  |
|  | > If ‘Antibiotics’: Current antibiotics types at the time of the visit | | | | Penicillins (such as penicillin, amoxicillin, co-amoxiclav, flucloxacillin and phenoxymethylpenicillin), Cephalosporins (such as cefalexin), Aminoglycosides (such as gentamicin and tobramycin), Tetracyclines (such as tetracycline, doxycycline and lymecycline), Macrolides (such as azithromycin, erythromycin and clarithromycin), Fluoroquinolones (such as ciprofloxacin and levofloxacin), Other, Unknown  > If ‘Other’: Specify other antibiotic types at the time of the visit | | Report the antibiotics types that are prescribed to the patient at the time of the visit. |  |
|  | Medication name and dose | | | |  | | Report the medication names and doses that are prescribed to the patient at the time of the visit.  Please report it in the following format (if information is available): *Medication name1 (generic name)_dose(units)_frequency(2d2t) *Medication name2 (generic name)_dose(units)_frequency(2d2t)  **2d2t meaning 2 times a day 2 tablets |  |
|  | Stopped medication types since the previous visit | | | |  | | Report the medication types that were stopped since the patient's previous visit. |  |
|  | > If ‘Antibiotics’: Stopped antibiotic types since the previous visit. | | | | > If ‘Other’: Specify other stopped antibiotic types at the time of the visit | |  |  |
|  |  | | | |  | |  |  |
