# Supplementary material for: The development of a data dictionary with clinical variables for artificial intelligence-driven tools in research on abdominal aortic aneurysms and peripheral arterial disease
Source: Eur Heart J Digit Health. 2025 Aug 20;6(6):1104–12. doi: 10.1093/ehjdh/ztaf091 (PMC12629649; doi:10.1093/ehjdh/ztaf091)
Supplement: ztaf091_Supplementary_Data [file ztaf091_supplementary_data.zip › Supplementary_Table_S3_Discussion_Points.docx]

**Supplementary Table S3: Discussion points and degree of consensus obtained during first and second consensus meetings. AAA = abdominal aortic aneurysm; PAD = peripheral arterial disease.**

| **Discussion points** | **Consensus** | **Reason for change** |
| --- | --- | --- |
| *General points* | | |
| A diagnosis of PAD or AAA should be made by a vascular surgeon or similarly qualified healthcare worker. | Consensus on inclusion | To ensure definitive diagnosis of PAD or AAA, eliminating potential uncertainties about diagnoses. |
| Suggestion to add the answer option “Do not know” to every question:  - “Do not know”: in medical file “unknown” is noted (e.g.: “ethnicity: unknown”)  - “Unknown”: information is missing from medical file (e.g. year of diagnosis cannot be found) | Consensus on **not** adding “Do not know” as answer option | A distinction between “Unknown” and “Do not know” is not relevant in our artificial intelligence model; these answer options would not be analyzed differently. |
| Ethnicity: suggestion to add Arabic, North African, Indian subcontinent, and (South-)East Asian to list of ethnicities (comprised of Asian, Black, Hispanic, Multiple ethnicity, White, Unknown, Other (specify)) | Suggestion to add a standardized ethnicity list from literature | To standardize the list of ethnicities, an evidence-based list from literature is needed. |
| Suggestion to add classification according to KDIGO (based on GFR and albuminuria) to variable asking presence of chronic kidney disease.  Options used to be: Yes / No / Unknown. | Consensus on including this variable.  Suggestion to add ‘If known’: consensus. | KDIGO is a known and frequently used classification system for chronic kidney disease. However, this may not be retrievable from electronic health records. Therefore, it is only reported when known. |
| Laboratory values: suggestion to remove thrombocytes, erythrocytes, MCV, MCH, MCHC, Urea | Consensus on removing all variables except thrombocytes. Thrombocytes are included in the data-dictionary. | The added value of erythrocytes, MCV, MCH, MCHC, and urea was disputed in the retrospective cohort studies. Moreover, consortium partners indicated that it would either not be reported or it would be very time-consuming to collect these values during data collection. Thrombocytes, however, are more frequently measured and were expected to be predictive in disease progression. |
| Laboratory values: suggestion to add urinary micro/creatinine ratio, vitamins, micronutrients | Consensus on **not** adding these variables. | The added value of these laboratory values was disputed and they would be difficult to collect in retrospective data collection. |
| Suggestion to add ‘Gestational diabetes’ as answer option to variable asking whether patient has diabetes mellitus.  Previous answer option were:   - No - DM 1 - DM 2, option to select whether DM2 is insulin-controlled - Other - Unknown | Consensus  Suggestion: to add ‘former’ or ‘current’ as answer option per diabetes type. Consensus obtained. | More encompassing answer options. Gestational diabetes was also deemed clinical relevant. |
| Suggestion to remove “Diastolic blood pressure usually <90 mmHg” and “If no: Blood pressure controlled (lower than 90 mmHg) with medicine?” and instead add variable “Hypertension mentioned in medical file as reported by treating physician” | Consensus | Cut-off points are often not recorded in this manner in retrospective data collection; therefore, clinical diagnosis was deemed sufficient. |
| Suggestion to change variable [hyperlipidemia] to “Hyperlipidemia mentioned in medical file by treating physician” | Consensus | Difficult to obtain exact laboratory data to objectively establish hyperlipidemia; therefore, clinical diagnosis was deemed sufficient. |
| Suggestion to remove pulmonary function tests (forced expiratory volume, pCO2, maximal voluntary ventilation) | Consensus | Difficult to obtain in retrospective data collection. |
| Instead of asking for degree of carotid stenosis, change variable to “Interventions performed for carotid stenosis” | Consensus | Degree of carotid stenosis is irrelevant if operation has occurred. |
| Suggestion to have “Atrial fibrillation” or “Atrial flutter” as answer options under “Atrial dysrhythmia” and all other atrial dysrhythmias to specify as open text under “Other” | Consensus | Minimizing answer options by having only the most frequently diagnosed atrial arrhythmias, to prevent an overabundant database. |
| Suggestion to add variable asking if patient has a pacemaker | Consensus | Gives an indication of degree of atrial arrhythmia. |
| *AAA data-dictionary* | |  |
| To change the answer options to the question in the AAA dictionary whether a patient has PAD to “None / Asymptomatic / Symptomatic without intervention / Invasive treatment for PAD (multiple answer options possible)” instead of simply “Yes / No / Unknown” | Consensus on **not** changing the definition  Suggestion during discussion: add “measured with a supervised walking test” after “maximum walking distance” > consensus on inclusion | The distinction between these subtypes may not be known in retrospective health records. |
| *PAD data-dictionary* | |  |
| If known, differentiation between treadmill test and anamnestic information should be made. We should ask:  - Maximum walking distance  - Anamnestic: … meters  As determined by treadmill test: … meters | Consensus on differentiating between treadmill test and anamnestic information.  Suggestion during discussion: to add supervised exercise therapy as answer option to | Anamnestic information may be less reliable, it is therefore important to make a distinction between objective and subjective values. |
| Neuropathy diagnosis may be made solely on clinical symptoms as defined (paresthesia and/or paralysis) instead of physical and/or neurological examination. | Consensus | Information on physical and/or neurological examination may be missing in retrospective data collection. |
| Suggestion to remove a variable that specified arterial location of pathology in patients with PAD. Instead, degree of pathology (stenosis) will be derived from imaging. | Consensus | Cut-off points were arbitrary and imaging analysis will reveal what degree of stenosis is clinically significant. |
| Reason for PAD intervention answer options change to (two suggestions were made):   1. Suggestion one   In case of intervention:   - Symptomatic (intermittent claudication) without prior exercise therapy - Symptomatic (intermittent claudication) despite prior exercise therapy - Rest pain without ulcer - Not healing wound/ ulceration   In case of re-intervention:   - Stenosis or occlusion of previously treated lesion - Stenosis or occlusion at different location then previously treated lesion  1. Suggestion two  - Disease progression - Reintervention because of unsuccessful previous intervention - Reintervention because of restenosis - Other - Unknown   Instead of (original answer options):   - Wounds that will not heal - Too many symptoms and more conservative treatments did not improve symptoms - Wound debridement - Reintervention because of unsuccessful previous intervention - Reintervention because of disease progression | Consensus on suggestion one | Standardized answer options that focus more on clinical signs of disease progression. |
| Suggestion to remove presence of Doppler sounds over limb arteries during physical examination from the data-dictionary. | Consensus | Difficult to obtain in retrospective data collection. |
| Suggestion to remove the following variables from physical examination findings:   - Cold feet - Discoloration of feet - Depending rubor - Edema | Consensus | Difficult to obtain in retrospective data collection and clinical relevance is disputed. |
| Suggestion to remove the variable whether multidisciplinary team (rehabilitation doctor, vascular internal medicine specialist, infectious disease specialist, podiatry, wound nurse) was involved in PAD treatment | Consensus | Difficult to obtain in retrospective data collection. |
